# Cryo-EM structures of CRAF/MEK1/14-3-3 complexes in autoinhibited and open-monomer states reveal features of RAF regulation

**DOI:** 10.1038/s41467-025-63227-2

**Published:** 2025-09-01

**Authors:** Dong Man Jang, Kayla Boxer, Byung Hak Ha, Emre Tkacik, Talya Levitz, Shaun Rawson, Rebecca J. Metivier, Anna Schmoker, Hyesung Jeon, Michael J. Eck

**Affiliations:** 1 Department of Cancer Biology, Dana-Farber Cancer Institute, Boston, MA 02215 USA; 2 Department of Biological Chemistry and Molecular Pharmacology, Harvard Medical School, Boston, MA 02115 USA; 3 Systems, Synthetic, and Quantitative Biology PhD Program, Harvard Medical School, Boston, MA USA

**Keywords:** Cryoelectron microscopy, Kinases, Phosphoproteins, Phosphorylation, Cancer

## Abstract

CRAF (RAF1) is one of three RAF-family kinases that initiate MAP kinase signaling in response to activated RAS and is essential for oncogenic signaling from mutant KRAS. Like BRAF, CRAF is regulated by 14-3-3 engagement and by intramolecular autoinhibitory interactions of its N-terminal regulatory region. Unlike BRAF, it is thought to require tyrosine phosphorylation in its N-terminal acidic (NtA) motif for full catalytic activation. Here we describe cryo-EM reconstructions of full-length CRAF in complex with MEK1 and a 14-3-3 dimer. These structures reveal a fully autoinhibited conformation analogous to that observed for BRAF and two “open monomer” states in which the inhibitory interactions of the CRD and 14-3-3 dimer are released or rearranged, but the kinase domain remains inactive. Structure-function studies of the NtA motif indicate that phosphorylation or acidic mutations in this segment increase catalytic activity by destabilizing the inactive conformation of the kinase domain. Collectively, these studies provide a structural foundation for understanding the shared and unique regulatory features of CRAF and will inform efforts to selectively block CRAF signaling in cancer.

## Abstract (web summary)

CRAF is one of three RAF-family kinases and is mutated in cancer and RASopathies. Jang et al. report structural and mechanistic studies of CRAF/MEK1/14-3-3 complexes that explain distinct features of CRAF regulation.

## Introduction

RAF family kinases ARAF, BRAF, and CRAF (Raf-1) are critical components of the RAS-RAF-MEK-ERK signaling pathway, which governs essential cellular processes including proliferation, differentiation, and survival^1–3^. This pathway is activated by growth factor receptors at the cell surface, leading to the activation of RAS proteins, which in turn recruit and activate RAF kinases^4^. Once activated, RAF kinases phosphorylate and activate MEK1/2, which subsequently phosphorylate and activate ERK1/2, culminating in the regulation of gene expression and cellular responses. The three RAF isoforms have both shared and distinct regulatory mechanisms^5^. BRAF has higher catalytic activity and is generally considered to have a simpler activation mechanism compared to CRAF and ARAF^6^. This difference may explain why BRAF mutations are prevalent in many types of cancers, whereas mutations in CRAF and ARAF are comparatively rare^7,8^.

**Fig. 1:**
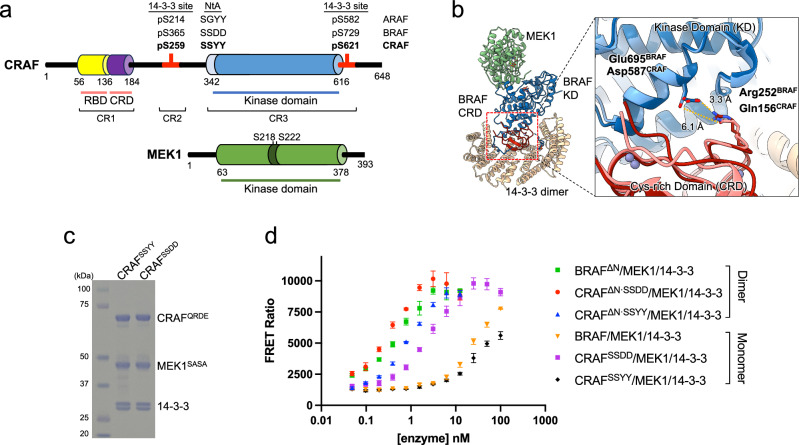
Domain organization, expression, and characterization of CRAF/MEK1/14-3-3 complexes. **a**, Schematic showing the domain organization of CRAF and MEK1. Residue numbers for domain boundaries are shown below and selected regulatory phosphorylation sites for ARAF, BRAF, and CRAF are indicated above the schematics. **b** Rationale for the engineered salt-bridge in CRAF. The autoinhibited BRAF/MEK1/14-3-3 complex (PDB ID: 6NYB) is superimposed on an AlphaFold3 model of an autoinhibited CRAF/MEK1/14-3-3 complex. The BRAF kinase domain and CRD are shown in dark blue and dark red, respectively, and the corresponding domains of CRAF in light blue and pink. The Q156R/D587E mutation in CRAF introduces a salt-bridge between the CRD and kinase domain analogous to the R252 to E695 salt-bridge observed in autoinhibited BRAF. **c** Coomassie-stained SDS-PAGE analysis of purified CRAF^SSYY^/MEK1/14-3-3 and CRAF^SSDD^/MEK1/14-3-3 complexes. The Q156R and D587E mutations (QRDE) are incorporated in both CRAF constructs. Staining is consistent with a stoichiometric complex of CRAF, MEK1, and a 14-3-3 dimer. Sf9 cells express two 14-3-3 isoforms, ε and ζ. The experiments were independently repeated twice with similar results. **d** Kinase activity of purified RAF complexes measured in a time-resolved (TR)-FRET-based biochemical assay (see *Methods* for details). The FRET ratio at 665/620 nm is plotted for increasing concentrations of each RAF protein. RAF dimers were generated using N-terminally truncated (ΔN) constructs lacking the RBD and CRD, while RAF monomers were obtained with full-length constructs. Data are presented as mean values ± standard deviation (SD); experiments were conducted three times independently, each time in triplicate, with similar results. Source data are provided as a Source Data file. NtA N-terminal acidic motif, RBD RAS-binding domain, CRD cysteine-rich domain, KD kinase domain, CR1-CR3 conserved regions 1-3.

RAF kinases share three conserved regions: CR1, CR2, and CR3 (Fig. 1a). CR1 contains the Ras-binding domain (RBD) and the cysteine-rich domain (CRD), which together interact with Ras and the plasma membrane to mediate membrane localization^1,9,10^. CR2 consists of a key autoinhibitory phosphorylation site (pSer259 in CRAF, pSer365 in BRAF) and CR3 contains the serine/threonine kinase domain. RAF is regulated by multiple mechanisms to ensure tight control of its catalytic activity. In its mature autoinhibited state, RAF forms a complex with two additional proteins, its substrate MEK1/2 and a 14-3-3 dimer^11–15^. 14-3-3 s are abundant proteins that participate in many signaling pathways via their ability to bind to specific phosphoserine- or phosphothreonine-containing motifs^16,17^. RAFs contain two 14-3-3 binding motifs, the CR2 site noted above and an additional site just C-terminal to the kinase domain. Structural studies of BRAF in its autoinhibited state have shown how a 14-3-3 dimer binds simultaneously to the CR2 and C-terminal (pSer365 and pSer729, respectively, in BRAF) recognition sites in a manner that blocks the ability of the kinase domain to dimerize, a key requirement for its activation^18,19^. The BRAF CRD domain stabilizes this autoinhibited state via interactions with the BRAF kinase domain, the 14-3-3 dimer, and both 14-3-3 binding sites. The RBD domain may also contribute to autoinhibition via interactions with 14-3-3, but unlike the CRD, which is shielded from interactions with the membrane, the RBD is largely exposed and accessible for binding to GTP-bound RAS^18,19^.

RAFs are activated by dimerization upon recruitment to the plasma membrane by GTP-bound RAS^10,20–23^. In contrast to the autoinhibited state in which the 14-3-3 blocks RAF dimerization, in the active state, the 14-3-3 dimer promotes dimerization by binding the C-terminal recognition sites of two RAFs, stabilizing formation of the active “back-to-back” dimer of two RAF kinase domains^18,19,24,25^. Activation also involves dephosphorylation of the CR2 14-3-3 binding site by the SHOC2 phosphatase complex, a ternary complex containing SHOC2, the RAS-related GTPase MRAS, and a protein phosphatase 1 catalytic subunit (PP1C)^26–31^. Dephosphorylation is thought to occur subsequent to “opening” of autoinhibited RAF at the plasma membrane, as the CR2 site is protected from dephosphorylation in the fully autoinhibited state^28,32^.

While the regulatory features above are common to all RAFs, the three isoforms differ in their requirements for phosphorylation on a site known as the N-terminal acidic motif (NtA), which lies immediately N-terminal to the kinase domain^33–35^. This site bears the sequence “SSDD” in BRAF, “SSYY” in CRAF, and “SGYY” in ARAF (Fig. 1a). In BRAF, one or both of the serine residues in this motif are constitutively phosphorylated. In CRAF and ARAF, full activation is thought to require phosphorylation at both the first serine and the last tyrosine within this motif. p21-Activated Kinases (PAK) and Protein Kinase C have been identified as responsible for phosphorylating the first serine, while Src family kinases phosphorylate the last tyrosine of the SSYY or SGYY motif^36–39^.

CRAF has long been known to mediate oncogenic signaling^40–42^. An early study by Blasco et al. highlighted the essential role of CRAF in the development of KRAS-driven non-small cell lung cancers, distinguishing it from other RAF family members like BRAF, which were found to be dispensable^42^. More recent work has extended these findings^43–46^. Interestingly, the kinase activity of CRAF is not required for its oncogenic activity, but its ability to dimerize is essential^45,46^. This is consistent with long-standing observations that mutant RAF proteins with impaired catalytic activity can nevertheless drive oncogenic transformation by dimerizing with catalytically competent RAF alleles and inducing their trans-activation^47^.

CRAF itself is recurrently mutated or activated as a fusion oncoprotein in cancer (~1%), though far less frequently than BRAF (~7%)^8,48^. The most common point mutations occur in the CR2 region (S257, S259, and P261), where they interfere with phosphorylation of S259 as required for 14-3-3-mediated autoinhibition^48^. Beyond cancer, numerous mutations in the RAF1 gene have been identified in Noonan Syndrome and other RASopathies, a spectrum of developmental syndromes caused by germline mutations in components of the RAS/MAP kinase pathway^49–51^. Like somatic mutations in cancer, these mutations also cluster in the CR2 region.

Despite intense interest in CRAF as a therapeutic target in cancer^52^, no structures are available for intact CRAF, and structures of its C-terminal region, including the kinase domain, have only recently emerged^53,54^. To better understand CRAF structure and regulation, including similarities and differences with respect to BRAF, we determined structures of full-length CRAF in complex with MEK1 and a 14-3-3 dimer using electron cryomicroscopy (cryo-EM). Single-particle reconstructions of this CRAF/MEK1/14-3-3 complex reveal three distinct structural states: i) an autoinhibited assembly analogous to that observed previously with BRAF, ii) an ”open monomer” state in which the CRD domain is released and interactions of the RAF/MEK kinase domain module with the 14-3-3 dimer are rearranged, and iii) a second open monomer in which there is no defined interaction of the RAF/MEK kinase module with the 14-3-3 dimer. In both open monomer states, the CRAF kinase domain is maintained in an inactive conformation but exposed for dimerization. Additionally, the pSer259 site is released from the 14-3-3 domain and exposed for dephosphorylation by the SHOC2 phosphatase complex in the open monomer states. Structure-function studies of the NtA regulatory motif suggest that this segment contributes to regulation by stabilizing the autoinhibited conformation of the RAF kinase domain in the absence of phosphorylation.

## Results

### Expression and characterization of the full-length CRAF/MEK1/14-3-3 complexes

Despite repeated attempts, we were unable to obtain a CRAF/MEK1/14-3-3 complex suitable for structural studies by co-expressing full-length wild-type CRAF with MEK1 in insect (Sf9) or mammalian cells due to low expression levels and protein aggregation during purification. Incorporation of a double Y340D/Y341D mutation in the CRAF NtA motif (which we refer to here as CRAF^SSDD^ because the SSYY sequence in CRAF is mutated to match the SSDD sequence of the corresponding region of BRAF, Fig. 1a) has been found to improve expression and increase catalytic activity of C-terminal fragments of CRAF^55,56^, but was not similarly enabling with full-length CRAF in our hands. In each of these efforts, we co-expressed a variant of MEK1 in which the activation loop is mutated (S218A/S222A, MEK1^SASA^) to prevent phosphorylation by RAF and thereby trap the complex^18^.

Sequence analysis and comparison of the structure of autoinhibited BRAF (PDB ID: 6NYB) with an AlphaFold-predicted model^57^ of autoinhibited CRAF indicated that a salt-bridge that stabilizes autoinhibited BRAF was not conserved in CRAF. This salt bridge in BRAF is formed between Arg252 in the CRD and Glu695 in the kinase domain (Fig. 1b). The corresponding residues in CRAF are Gln156 and Asp587. With the goal of enhancing the expression and stability of CRAF, we engineered this salt bridge into CRAF with a double Q156R/D587E mutation. We incorporated this salt bridge both in the context of the wild-type NtA motif (CRAF^SSYY^) and with the CRAF^SSDD^ variant to obtain CRAF^SSYY·QRDE^ and CRAF^SSDD·QRDE^. Co-expression of CRAF^SSYY·QRDE^ or CRAF^SSDD·QRDE^ with MEK1^SASA^ in Sf9 insect cells allowed co-purification of the corresponding CRAF variant with MEK1^SASA^ and an endogenous 14-3-3 dimer derived from the insect cells (Fig. 1c**)**. On size-exclusion chromatography, these CRAF complexes eluted at a volume consistent with a “monomeric” complex – i.e., a single chain of each component, as expected for the autoinhibited state based on prior work with BRAF (Supplementary Fig. 1). Because we incorporate the Q156R/D587E mutation in all of the full-length CRAF and the S218A/S222A mutation in the MEK1 constructs used in our structural studies, we omit the “QRDE” and “SASA” superscript notations from hereon.

To compare the kinase activity of these full-length CRAF/MEK/14-3-3 complexes with that of other RAF complexes, we prepared the full-length autoinhibited BRAF complex (BRAF/MEK1/14-3-3) and N-terminally truncated dimeric CRAF and BRAF complexes as previously described^56^. The N-terminal truncations remove the RBD and CRD and therefore yield active, dimeric complexes (CRAF^308-648^/MEK1/14-3-3, BRAF^419-766^/MEK1/14-3-3)^56^. We measured the kinase activity of these constructs using a time-resolved FRET (TR-FRET) assay that reports on phosphorylation of an exogenous wild-type MEK1 substrate (on S218/S222)^56^. As anticipated, the full-length BRAF and CRAF^SSYY^ complexes exhibit a low level of activity, presumably because they are monomeric and adopt an autoinhibited conformation (Fig. 1d**)**. By contrast, the dimeric complexes were highly active, with the CRAF^SSDD^ dimer exhibiting the highest activity, followed by dimeric BRAF and CRAF^SSYY^ (Fig. 1d). Interestingly, the monomeric CRAF^SSDD^ complex was also highly active, approaching that of the dimeric N-terminal deletion complexes (Fig. 1d). Activity assays of freshly collected size-exclusion fractions revealed increased catalytic activity in fractions containing the monomeric CRAF^SSDD^ complex, suggesting that the Y340D/Y341D mutation in the CRAF NtA motif can at least partially activate CRAF in its monomeric state (Supplementary Fig. 2a–d). To further probe whether CRAF^SSDD^ is dependent on dimerization for its catalytic activity, we prepared a CRAF^SSDD^ complex with an R401H mutation in the CRAF kinase domain (CRAF^SSDD, R401H^/MEK1/14-3-3). This mutation corresponds to the R509H substitution in BRAF and has been previously shown to prevent dimerization-driven activation of CRAF^58^. While the CRAF^SSDD, R401H^ complex was modestly more active than the CRAF^SSYY^ complex, it was far less active than the corresponding CRAF^SSDD^ complex, confirming that CRAF^SSDD^ remains dependent on dimerization for the bulk of its activity (Supplementary Fig. 2e). Taken together, these results suggest that the activity of the CRAF^SSDD^ complex arises from transient dimerization that is not evident on size-exclusion chromatography.

### Cryo-EM structures of CRAF/MEK1/14-3-3 complex in autoinhibited and open-monomer states

**Fig. 2:**
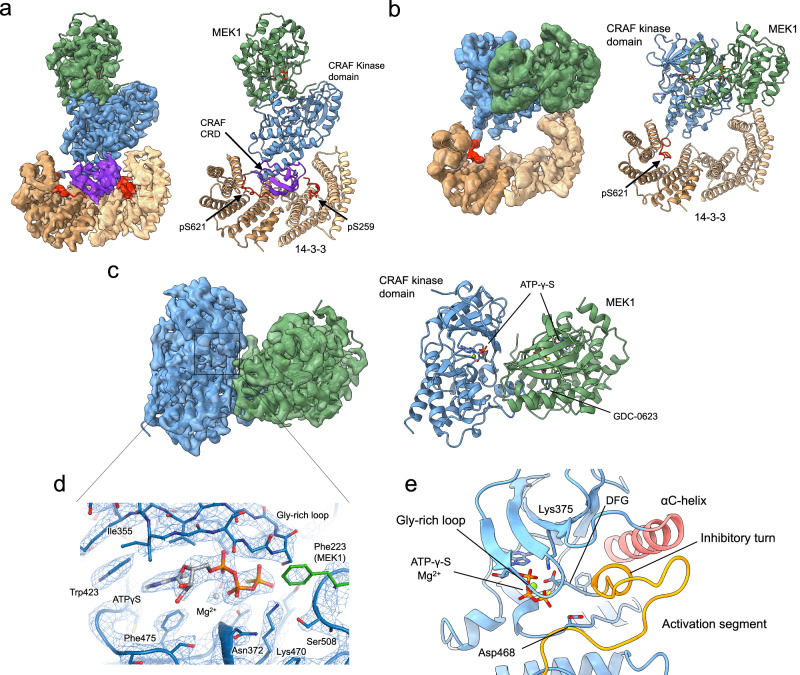
Cryo-EM structures of CRAF/MEK1/14-3-3 complexes in autoinhibited and open monomer states. Overall architectures of CRAF/MEK1/14-3-3 complexes in **a** the autoinhibited configuration, **b** the open monomer configuration, and (**c**) the kinase domain open monomer configuration. Cryo-EM maps in **a** and **c** were obtained with the CRAF^SSYY^ complex, the open monomer map in **b** was obtained with the CRAF^SSDD^ complex. Structures are shown in ribbon representations with (left) and without (right) their respective transparent cryo-EM density maps and are colored as in Fig. 1a. ATP analog ATPγS and MEK inhibitor GDC-0623 are depicted in stick representation. **d** A close-up view of the active-site cleft of CRAF, overlaid with the cryo-EM density map at 2.3 Å resolution. **e** Detailed view of the inactive conformation of the CRAF kinase domain. The C-helix (red) is stabilized in an outward, inactive conformation by the inhibitory turn within the activation segment (orange). Selected active site residues are shown in stick form.

We imaged monomeric fractions of both CRAF^SSDD^/MEK1/14-3-3 and CRAF^SSYY^/MEK1/14-3-3 complexes using single particle cryo-EM. Both complexes were stabilized by the addition of ATPγS and MEK inhibitor GDC-0623. To our surprise, ab-initio 3D reconstructions revealed three distinct conformational states in both the CRAF^SSYY^ and CRAF^SSDD^ complexes; an autoinhibited conformation closely resembling that we observed with BRAF (Fig. 2a) and two “open monomer” states in which inhibitory interactions of the kinase domain with the CRD and 14-3-3 are released or rearranged. We refer to these as open monomer states because they contain a single CRAF chain and retain an inhibited conformation of the CRAF kinase domain, in contrast to active CRAF/14-3-3 complexes that contain two CRAF kinases that form the active kinase dimer. In one of these open monomer states, the CRAF and MEK1 kinase domains adopt a defined orientation with respect to the 14-3-3 dimer (Fig. 2b). In the other there is no-defined orientation with respect to the 14-3-3, allowing reconstruction of the “isolated” CRAF/MEK1 kinase domain module (Fig. 2c). We refer to this configuration as the “kinase domain open monomer” complex.

We describe here single-particle reconstructions and models for a total of four structures derived from four datasets recorded from three cryo-EM grids (Supplementary Figs. 3–7, Supplementary Tables 1 and 2). Although the autoinhibited conformation and both open monomer states were present in each dataset, we describe here only the best-resolved structures for the autoinhibited and open monomer states, which were determined with the CRAF^SSYY^ and CRAF^SSDD^ datasets, respectively. For the kinase domain open monomer, we describe structures determined with both CRAF^SSYY^ and CRAF^SSDD^ preparations. Datasets used for the CRAF^SSDD^ kinase domain open monomer and CRAF^SSDD^ open monomer were recorded from the same grid, the latter at a 30° tilt to mitigate the effects of a preferred orientation of these particles.

In the fully autoinhibited structure, determined at 3.4 Å resolution, the phosphorylated pSer259 and pSer621 sites that flank the CRAF kinase domain are bound in recognition grooves on opposite sides of the 14-3-3 dimer (Fig. 2a). The CRD is nestled in the cradle of the 14-3-3 dimer, where it contacts both protomers of the 14-3-3, both phosphoserine segments, and the C-lobe of the CRAF kinase domain. Arg156 in the CRD, introduced with the Gln156Arg substitution, is positioned to form a salt-bridge with mutant Glu587 in the kinase domain as predicted (Supplementary Fig. 8b). While this engineered salt-bridge likely stabilizes the autoinhibited state, these mutations do not appear to affect interactions in the open monomer states described below. A prior mutagenesis study identified 11 substitutions in the CRAF CRD domain that led to increase CRAF activity^59^. All 11 of these residues participate in interdomain interactions in the autoinhibited structure and one, Arg143, hydrogen bonds with Gln15 and is the site of rare but recurrent mutations in cancer (Supplementary Fig. 8a, c). While there was insufficient density to allow modeling of the RBD, a reconstruction low-pass filtered to 6 Å and contoured at a lower threshold showed additional density corresponding to the RBD adjacent to the CRD (Supplementary Fig. 8d). The disorder of the RBD is reminiscent of BRAF, where this domain is variably ordered in the autoinhibited state^18,19^.

The CRAF kinase domain exhibits a characteristic “C-helix-out” inactive conformation and is oriented with its active site facing away from the 14-3-3, where it is closely juxtaposed with MEK1. MEK1 also adopts its characteristic inactive conformation as expected. This mutually autoinhibited RAF/MEK kinase domain module is essentially identical to that of the kinase domain open monomer as we describe in more detail below. Overall, the autoinhibited CRAF conformation is closely similar to that of autoinhibited BRAF (PDB ID: 6NYB) and the two structures superimpose with a root-mean-square deviation (RMSD) of 1.5 Å over 1074 aligned Cα atoms (Supplementary Fig. 9a). The interaction networks involving pSer259 in CRAF and pSer365 in BRAF with the CRD and 14-3-3 are largely conserved (Supplementary Fig. 9b). However, comparison of the two structures reveals a few significant differences. Structure-based superposition aligned on RAF reveals that MEK1 is rotated by approximately 5° in the CRAF structure, but in a manner that maintains key interactions with the CRAF kinase domain (Supplementary Fig. 9c). The MEK1 activation loop makes a short anti-parallel β-strand interaction with the CRAF activation loop centered on Phe223 in MEK1 and Ser508 in CRAF, and this structural element is shifted toward the CRAF C-lobe as compared with its position in the BRAF autoinhibited structure. However, this alternate conformation has been seen previously in a subset of BRAF/MEK crystal structures^60^. Finally, the interface between the CRD and 14-3-3 appears to be somewhat more tightly packed in BRAF than in CRAF. The BRAF contact buries 4,803 Å^2^ of surface area for 14-3-3, while the CRAF buries 4,435 Å^2^. This difference is due in part to two phenylalanine residues in BRAF (Phe243 and Phe256) that are replaced by leucine in CRAF (Leu147 and Leu160) near the CRAF pSer259 phosphosite (Supplementary Fig. 9b). This difference may account in part for the apparent instability of the autoinhibited state in CRAF as compared with BRAF. In all four CRAF cryo-EM datasets analyzed here, particles corresponding to the open monomer states were far more abundant than those representing the fully autoinhibited complex (Supplementary Figs. 3–6).

As noted above, we observed two open monomer configurations on the same EM grids with the fully autoinhibited complex. While an open monomer state has been hypothesized for RAFs, to our knowledge it has not been previously observed^3,32^. The CRAF^SSDD^/MEK1/14-3-3 open monomer cryo-EM map has a nominal resolution of 3.9 Å (unmasked), and despite use of a 30° tilt in data acquisition, still suffers somewhat from missing orientations (Supplementary Table 2, Supplementary Fig. 7c). Nevertheless, the map clearly reveals key structural features – the CRAF pSer621 phosphosite is bound on one side of the 14-3-3 dimer, and the MEK1 kinase domain contacts the opposite protomer of the 14-3-3 (Fig. 2b). The phosphopeptide recognition groove on this side of the 14-3-3 is unoccupied, confirming that the CRAF pSer259 motif is not engaged in this state (Fig. 2b). Notably, there is a visible gap between the CRAF and MEK1 kinase domains and the 14-3-3 dimer with no apparent density for the CRD, indicating that it is released and does not interact with 14-3-3 in the open monomer state. (Fig. 2b). The 14-3-3 contacts the C-lobe of MEK1 in a loop region (MEK1 residues 238-241) and makes a glancing contact at Pro346 (Supplementary Fig. 10a).

Single-particle reconstruction of the kinase domain open monomer yielded a high-resolution reconstructed map (2.9 Å unmasked, 2.3 Å masked) for the CRAF^SSYY^/MEK1 complex (Fig. 2c), which enabled us to model ligands including ATP analogs and the allosteric MEK inhibitor (Fig. 2d**and** Supplementary Fig. 10b). It appears that the 14-3-3 dimer still associates with the pSer621 site in the C-terminus of CRAF in this state, but due to the highly variable orientation of the 14-3-3 dimer, particles are aligned based on the larger CRAF/MEK1 kinase module, resulting in a 3D reconstruction that reveals only the CRAF and MEK1 kinase domains (Supplementary Fig. 11). This interpretation is further supported by counterexamples; we observed a few 2D classes where particles aligned based on the 14-3-3 dimer, causing the CRAF-MEK1 kinase monomer not to align properly and appear blurry (Supplementary Fig. 11).

The conformation of the CRAF kinase domain in the autoinhibited state is closely similar to that observed in autoinhibited BRAF^3^. The activation loop forms an autoinhibitory turn that locks the C-helix in an outward inactive position (Fig. 2e). As in BRAF, binding of ATP or a close analog appears to be important for stabilization of this closed, inactive conformation. Key residues that stabilize this state are conserved between CRAF and BRAF, including Val492 and Lys493 in the inhibitory turn (corresponding to Val600 and Lys601 in BRAF) and Phe360 in the P-loop (corresponding to Phe468 in BRAF, see Supplementary Fig. 12a, b). The inactive conformation of MEK1 is essentially identical to that seen in previous structures of MEK1 bound to RAF kinase domains^18,19,54,60^, as are the interactions between CRAF and MEK1 – the C-lobe of the two kinases interact via their αG helices in the C-lobe, a short antiparallel β-strand interaction formed by their activation loops, and an N-lobe contact in which Glu102 in MEK1 is positioned to hydrogen bond with the ribose of the ATPγS bound to CRAF (Supplementary Fig. 12c). As noted above, these interactions are maintained despite at 5° rotation of MEK1 relative to its position in autoinhibited BRAF structures. We also obtained a kinase domain open monomer reconstruction for the CRAF^SSDD^ complex, and the resulting structure was essentially the same as that obtained with the CRAF^SSYY^ complex (RMSD = 0.42 Å over 524 aligned Cα atoms). While the nominal resolution of the CRAF^SSDD^ kinase domain open monomer map was 3.3 Å (unmasked, 2.8 Å masked), the level of structural detail was somewhat less than could be expected at this resolution due to missing/poorly represented orientations for this particle class (Supplementary Fig. 7d).

### The open monomer state is poised for activation

**Fig. 3:**
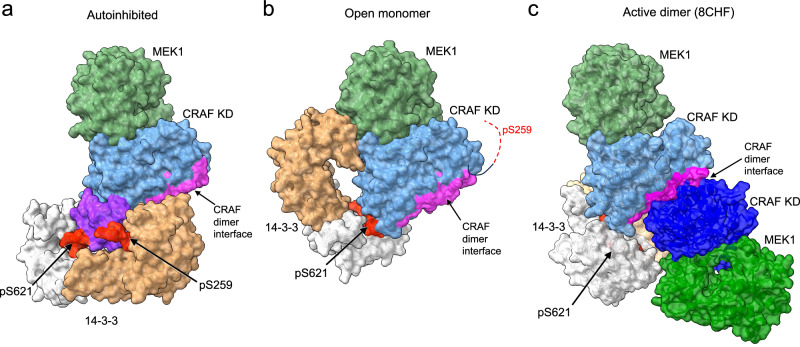
The open monomer state is poised for activation. The comparison of the autoinhibited (**a**), open monomer (**b**), and active dimer (**c**) states of the CRAF/MEK1/14-3-3 complex. Structures are shown in a surface representation and with the same orientation of the CRAF and MEK1 kinase domains. Residues of the CRAF kinase domain that form the dimer interface in active CRAF dimers is shaded violet in each structure. **a** In the autoinhibited state, the 14-3-3 blocks the CRAF dimer interface and also sequesters the CRD (purple). **b** In the open monomer state, the pSer259 segment and the CRD are released from the 14-3-3 and are therefore not visible in the cryo-EM maps. The pSer621 site remains engaged with the 14-3-3 dimer, which has reoriented to expose the CRAF dimer interface. **c** In the active, dimeric state, the 14-3-3 dimer engages the pSer621 sites of two CRAFs, stabilizing the activating back-to-back interaction of the CRAF kinase domains. The active dimer is drawn from a previously reported cryo-EM structure (PDB ID: 8CHF) that was obtained with a truncated CRAF construct lacking the RBD, CRD and pSer259 regions.

The direct mechanism of RAF activation is the formation of the back-to-back dimer of the kinase domain, which rearranges the C-helix to its inward active position to complete the kinase active site^61^. Activation is promoted by dephosphorylation of the pSer259 site, which is thought to favor dimerization and activation by precluding return to the fully autoinhibited state, which requires phosphorylation on both Ser259 and Ser621^3,27^. In the fully autoinhibited state of CRAF, the kinase domain is protected from dimerization by the 14-3-3 dimer, and the pSer259 site is expected to be inaccessible for dephosphorylation by the SHOC2 phosphatase complex (Fig. 3a).

The structures of the CRAF open monomer complexes show that both of these hindrances to activation are removed in this state. As compared with the intact autoinhibited state, the 14-3-3 domain in the open monomer is re-oriented to expose the dimerization interface of the kinase domain (Fig. 3b, c). Although the 14-3-3 remains bound to the pSer621 site, it rotates by ~180° and shifts by ~35 Å such that it no longer sterically occludes the kinase dimerization interface. Obviously the dimerization interface is also fully exposed in the CRAF/MEK1 kinase domain open monomer, in which the 14-3-3 has no defined orientation with respect to the kinase domain. We do not ascribe a particular significance to the open monomer state as compared with the kinase domain open monomer; in both the CRD and pSer259 interactions are released and the CRAF kinase domain is exposed for dimerization.

**Fig. 4:**
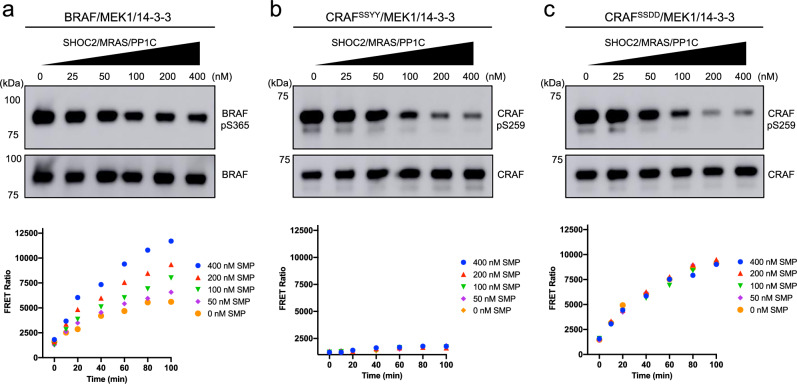
Differential responses of RAF proteins to dephosphorylation by the SHOC2-MRAS-PP1C complex. In vitro dephosphorylation of autoinhibited BRAF/MEK1/14-3-3 (**a**), CRAF^SSYY^/MEK1/14-3-3 (**b**), and CRAF^SSDD^/MEK1/14-3-3 (**c**) complexes by the SHOC2-MRAS-PP1C holophosphatase complex. The purified full-length RAF complexes were incubated with increasing concentrations of the SHOC2-MRAS-PP1C complex. Aliquots of the dephosphorylation reactions were analyzed for phosphorylation at pSer365 (BRAF) or pSer259 (CRAF) by Western blotting with a phosphospecific antibody for this site and for total BRAF or CRAF as a loading control (upper panels). The kinase activity of aliquots of the dephosphorylation reactions was assessed using a TR-FRET-based assay (lower panels). Dephosphorylation reactions were stopped by addition of a phosphatase inhibitor and samples were then diluted in reaction buffer for the kinase assay. The FRET ratio at 665/620 nm is plotted over the time course upon addition of the reaction mixture containing wild-type MEK1 as a substrate and ATP. Each data point represents the mean of triplicates from one representative experiment. The experiments were independently repeated twice with similar results. Source data are provided as a Source Data file.

The open monomer structures also indicate that the pSer259 site is exposed for dephosphorylation in this state. Mass spectrometry confirms that CRAF Ser259 is phosphorylated to a high stoichiometry in our protein preparations (Supplementary Table 3), but there is no density visible for this phosphosite in the binding cleft of the 14-3-3 domain in the open monomer. To test accessibility pSer259 to dephosphorylation, we examined dephosphorylation of this site and compared it with dephosphorylation of the corresponding site in BRAF (pSer365), which we previously found to be relatively resistant to dephosphorylation in the autoinhibited state (as compared with the active dimeric state in which this site in not engaged with the 14-3-3)^28^. We incubated the autoinhibited CRAF/MEK/-14-3-3 and BRAF/MEK/14-3-3 preparations with increasing concentrations of the SHOC2-MRAS-PP1C complex and measured the remaining levels of CRAF pSer259 or BRAF pSer365 by western blotting with a phospho-specific antibody directed against the pSer259/pSer365 site (Fig. 4a–c). Consistent with our prior studies^28^, we observed a modest degree of dephosphorylation of BRAF pSer365 **(**Fig. 4a**)**. By comparison, the CRAF pSer259 site was more completely dephosphorylated in both the CRAF^SSDD^/MEK1/14-3-3 and CRAF^SSYY^/MEK1/14-3-3 complexes (Fig. 4b, c).

We next tested the effect of SHOC2-mediated dephosphorylation of these RAF complexes on their kinase activity. To do this, we sampled CRAF and BRAF proteins after dephosphorylation by increasing concentrations of the SHOC2-MRAS-PP1C complex and measured MEK1 or ERK2 phosphorylation levels in the presence of ATP and phosphatase inhibitors (Fig. 4a–c, lower panels and Supplementary Fig. 13**)**. We observed that BRAF kinase activity significantly increased with higher SHOC2-MRAS-PP1C concentrations, correlating with dephosphorylation of pSer365 (Fig. 4a and Supplementary Fig. 13). This indicates that although pSer365 is not efficiently dephosphorylated by the SHOC2-MRAS-PP1C complex, BRAF transitions into its active conformation upon dephosphorylation of this site. CRAF, however, behaved differently. We did not observe a significant change in CRAF activity following dephosphorylation by the SHOC2-MRAS-PP1C complex. The CRAF^SSYY^ complex exhibited little kinase activity and the CRAF^SSDD^ complex remained similarly active despite a substantial degree of dephosphorylation of pSer259 in both proteins (Fig. 4b,c and Supplementary Fig. 13). While this lack of catalytic activation may seem surprising, it is consistent with the notion that dephosphorylation of this site favors activation by increasing the abundance of open monomers (by precluding the kinase domain from adopting the fully autoinhibited state), rather than by directly driving formation of active dimers. Given that a majority of molecules in our CRAF preparation are already in the open monomer state and do not spontaneously convert to active dimers, there is no reason to expect that dephosphorylation of pSer259 would result in catalytic activation.

### The role of NtA motif in CRAF regulation

**Fig. 5:**
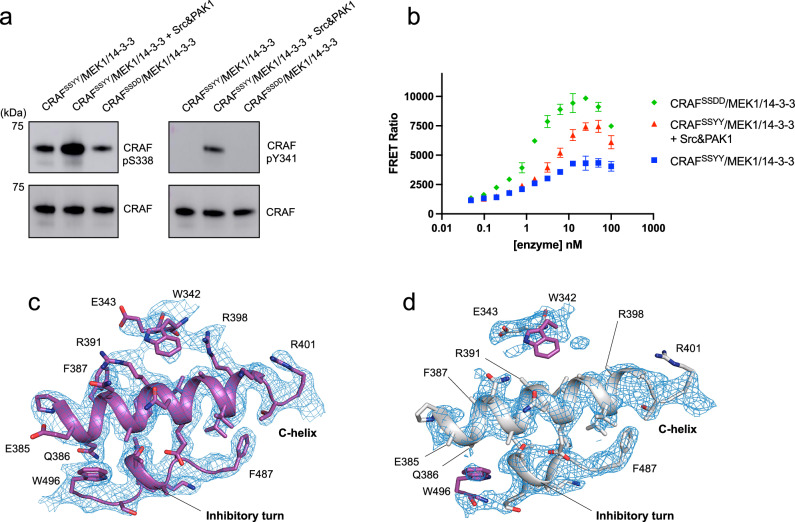
Effects of NtA motif phosphorylation or mutation on structure and activity of CRAF. **a** Full-length CRAF^SSYY^ and MEK1 were co-expressed in Sf9 cells with or without accompanying co-expression of activated Src and PAK1. After purification, the phosphorylation status of the NtA motif of the resulting CRAF^SSYY^/MEK1/14-3-3 complexes was assessed by western blotting with phosphospecific antibodies directed against pSer338 (Cell Signaling Technology, #9427) and pTyr341 (Abcam, #ab59223) (upper panels) and against total CRAF as a loading control (lower panels). **b** Kinase activity of CRAF^SSDD^/MEK1/14-3-3 and CRAF^SSYY^/MEK1/14-3-3 complexes prepared with or without co-expression of activated Src and PAK1 was assessed in the TR-FRET assay. The FRET ratio at 665/620 nm is plotted for increasing concentrations of each RAF complex. Each data point represents the mean ± SD of triplicate measurements. The decrease in activity at high enzyme concentrations is due to competition from the non-phosphorylatable MEK1^SASA^ in the enzyme complex, which becomes significant as its concentration approaches that of the WT MEK1 substrate (200 nM). Density maps in the region of the C-helix in cryo-EM structure of the CRAF^SSYY^/MEK1/14-3-3 kinase domain open monomer (**c**) and the crystal structure of CRAF^SSDD^ and MEK1 kinase domains (PDB ID: 9AY7) (**d**). In the CRAF^SSYY^ structure in **c**, there is clear side chain density for residues in the C-helix and inhibitory turn, and for W496 in the activation loop and W342, the first residue following the NtA motif. By contrast, there is evidence of local disorder in this region in the crystal structure in (**d**). W342 and W496 are not modeled in the structure and are superimposed from the CRAF^SSYY^ structure (and colored purple) to indicate their positions. In addition, several large residues in this region of the CRAF^SSDD^ structure were modeled as alanine owing to a weak or no density for their sidechains (including Q386, F387, R391, and R398). The density map for the cryo-EM structure is shown in **c**, and the 2*Fo* − *Fc* map for the crystal structure in **d** is contoured at 1.0 σ.

Although CRAF has long been known to be activated by phosphorylation on Ser338 and Tyr341 in its NtA motif (-^338^SSYY^341^-)^33–35^, to our knowledge the effect of these phosphorylations has not been studied with purified full-length CRAF in vitro. To explore this further, we co-expressed CRAF and MEK1 with activated variants of PAK1 (PAK1 T423E) and c-Src (c-Src Y530F), which are reported to phosphorylate Ser338 and Tyr341 in the CRAF NtA motif, respectively^36–39^. After purification of the CRAF/MEK1/14-3-3 complex, we assessed phosphorylation of the SSYY motif by western blotting with phospho-specific antibodies directed against pSer338 and pTyr341. With the pSer338-directed antibody, we observed robust reactivity with the CRAF^SSYY^ and CRAF^SSDD^ complexes prepared without co-expression of Src and PAK1, but reactivity was markedly greater in the CRAF complex prepared by co-expression with Src and PAK1 (Fig. 5a). Blotting with the pTyr341-specific antibody revealed phosphorylation of this tyrosine only in the CRAF complex prepared by co-expression with Pak1 and Src (Fig. 5a). The catalytic activity of this Src/PAK1-phosphorylated CRAF^SSYY^ complex was higher than that of the corresponding complex prepared without Src and PAK1 co-expression, but markedly less than that of the CRAF^SSDD^ complex (Fig. 5b). To date we have not been able to quantitate this Tyr341 phosphorylation using mass spectrometry, so it is unclear whether the lower level of activity arises from a low stoichiometry of phosphorylation or whether the SSDD mutant is hyperactivated as compared with the tyrosine-phosphorylated protein.

Phosphorylation of the NtA motif is thought to activate CRAF by promoting dimerization, and multiple studies report increased dimerization/heterodimerization by the CRAF^SSDD^ phospho-mimetic mutant^7,55,62^. One model suggests that the phosphorylated segment interacts in trans with a basic patch on N-lobe of the kinase^55,62,63^. Phosphorylation or mutation of the NtA motif could promote activation via one or both of two general mechanisms – destabilizing the inactive conformation of the kinase to activate the monomer and/or directly stabilizing the active dimeric state. The former could also favor dimer formation by lowering the energetic barrier to dimerization. Notably, the proportion of fully autoinhibited particles is markedly less in our CRAF^SSDD^ dataset as compared with the CRAF^SSYY^ dataset (Supplementary Figs. 3–5), but we do not observe 2D or 3D classes corresponding to active dimers. We also had the subjective impression that the kinase domain open monomer particles were more heterogeneous in the CRAF^SSDD^ dataset; these particles resolved into multiple classes in early stages of processing in the CRAF^SSDD^ dataset as compared with a single class in the CRAF^SSYY^ dataset (Supplementary Figs. 3–5). However, we were only able to reconstruct a single conformation from the CRAF^SSDD^ images and as noted above, the resulting structure was essentially the same as that obtained with the CRAF^SSYY^ images.

The NtA motif is not resolved in any of our structures, and to our knowledge, it has not been observed in any previously reported RAF structure, active or inactive. This motif is immediately N-terminal to Trp342, a residue that is conserved as a large hydrophobic amino acid in many kinases that are regulated by C-helix movements. In Src-family kinases, the corresponding tryptophan interdigitates between the C-helix and the β4 strand in the N-lobe to help maintain the inactive C-helix out conformation^64^. Trp342 in our CRAF^SSYY^ structure assumes the corresponding position, and the C-helix and adjacent inhibitory turn portions of the activation segment are clearly resolved (Fig. 5c). By contrast, these regions are poorly resolved and exhibit high B-factors in a recently reported inactive-state crystal structure of CRAF^SSDD^ kinase domain with MEK (Fig. 5d)^54^. As described above, the SSDD substitution increases the catalytic activity of monomeric CRAF complexes, but has little effect in the context of 14-3-3-bound CRAF dimers (Fig. 1d). Although the CRAF^SSDD^ complexes do not form stable dimers, the effect of the R401H mutation indicates that they rely on transient kinase domain dimerization for the bulk of their catalytic activity (Supplementary Fig. 2e). Collectively, these observations and those above lead us to propose that the NtA motif contributes to CRAF regulation by helping to stabilize the “C-helix-out” inactive conformation of the CRAF kinase domain. Phosphorylation of the native SSYY sequence or its mutation to SSDD promotes activation by destabilizing this state, leading to increased catalytic activity via an increased propensity to dimerize.

## Discussion

We have used cryo-EM to determine the first structures of intact CRAF in complex with MEK1 and a 14-3-3 dimer, with a focus on the autoinhibited state. Together with recently reported structures of C-terminal fragments of CRAF in the active dimeric state^53^, these structures reveal the outlines of CRAF regulation. In the autoinhibited state, the 14-3-3 dimer binds the pSer259 and pSer621 sites of one CRAF kinase, protecting it from dimerization and also sequestering the CRD to prevent membrane-localization in the absence of RAS activation. By contrast, in the active state, a 14-3-3 dimer binds the pSer621 site of two CRAFs, bringing them together to form the active back-to-back dimer^53^. Unexpectedly, we found that a large fraction of the CRAF complexes in our preparations assume one of two open monomer configurations in which the pSer259 site and CRD are released from the 14-3-3 dimer (Fig. 2b, c). Open monomer states have not been previously structurally characterized for any RAF. Autoinhibited, open monomer and active dimer states of CRAF are depicted in Fig. 3.

We propose that the open monomer state represents an intermediate between the closed, autoinhibited state and the active dimer configuration. Although the kinase domain remains in the inactive conformation in the open monomer, the RBD and CRD are free to interact with active RAS at the membrane, restraints on the 14-3-3 dimer are released such that it can rearrange to engage the pSer621 site of two RAFs, and the steric block of the dimerization surface of the CRAF kinase domain by the 14-3-3 domain is removed. In addition, the pSer259 becomes exposed such that it can be readily dephosphorylated by the SHOC2 phosphatase complex. MEK1 adopts essentially the same conformation in the autoinhibited and both open monomer states, with its activation segment forming a helix that would appear to prevent ready access of Ser218 and Ser222 to the RAF active site. It remains a puzzle how this inhibitory feature may be released, but it is possible that the helix is unstable and that transient unfurling is sufficient to allow its phosphorylation by RAF.

It is unclear why the open monomer states do not spontaneously progress to form active dimers in our preparation. As with BRAF, binding of ATP to the CRAF kinase domain stabilizes its inactive conformation (Fig. 2e), disfavoring dimer formation, but we would expect 14-3-3-driven dimerization to overcome this effect as described for BRAF^25^. One possibility is that this is a limitation of our in vitro system. In cells, the higher local concentration of open monomers engaged with active RAS at the membrane would be expected to favor dimerization. CRAF may also be intrinsically less prone to homodimerize, which could explain its lower catalytic activity in the absence of phosphorylation of its NtA motif as compared with BRAF. We have not explored whether CRAF would more readily form active heterodimers with BRAF or ARAF. This is an important area for further study, as heterodimerization is a prominent feature in RAF activation^1,65–68^.

Obtaining sufficient CRAF complex suitable for cryo-EM imaging required the introduction of a salt-bridge designed to stabilize the autoinhibited state. This salt bridge lies in the interface between the CRD and kinase C-lobe, and mimics one found in BRAF but not conserved in CRAF and ARAF. Unlike BRAF, CRAF is known to depend heavily on the HSP90/CDC37 chaperone complex^69–71^. Garcia-Alonso et al. have recently determined cryo-EM structures of CRAF bound to HSP90 and CDC37, showing how the CRAF kinase domain is engaged with the chaperone in a partially unfolded state^72^. By stabilizing the 14-3-3-bound autoinhibited state, the engineered salt bridge may decrease the proportion of CRAF engaged with HSP90/CDC37, yielding more of the fully folded and assembled complex with MEK1 and 14-3-3.

The preponderance of the open monomer in our preparations is consistent with recent work showing that CRAF is less tightly regulated by CRD-mediated autoinhibition as compared with BRAF^73^. Despite the abundance of the open monomer, it is clear that the fully closed autoinhibited state is relevant to CRAF regulation – most oncogenic and rasopathy mutations in CRAF perturb phosphorylation of the S259 14-3-3 binding site^48,51^, and truncations of the CRD region have long been known to result in CRAF activation^74^. Thus, it appears that CRAF is simply less tightly regulated via this mechanism as compared with BRAF.

RAS-driven membrane recruitment and phosphorylation of the NtA motif by Src kinases can independently activate CRAF, but both are required for maximal activation^34,75,76^. Beyond a role as an intermediate in RAS-dependent activation, the open monomer state may set the stage for this two-part regulatory mechanism. We speculate that the open monomer state, as opposed to the fully autoinhibited state, is a substrate for NtA phosphorylation. Because we have not been able to separate open monomers from fully autoinhibited CRAF/MEK/14-3-3 complexes, we have not tested this notion directly. However, phosphomimetic mutations in the CRAF NtA have previously been shown to interfere with autoinhibition mediated by the N-terminal RBD/CRD region^77^, and consistent with this our CRAF^SSDD^ preparations have a very low fraction of particles in the fully autoinhibited state (Supplementary Fig. 5). And while open monomers that are phosphorylated on the NtA motif (or that bear the SSDD mutation) are catalytically active, they are less active than CRAF dimers (Fig. 1d). Thus it is reasonable to expect that in a cellular context, NtA-phosphorylated open monomers could be further activated by RAS-driven, 14-3-3 stabilized dimerization at the plasma membrane.

The phosphorylated NtA motif has been suggested to promote kinase domain dimerization and activation via binding in trans to the opposite protomer^55,62,63^. While we do not exclude a possible contribution to activation via this mechanism, our data suggest that phosphorylation of this segment or its replacement with the SSDD sequence found in the corresponding region of BRAF destabilizes the autoinhibited conformation of the kinase domain in a manner that involves repositioning of Trp342. Trp342 has previously been shown to be important for stabilizing RAF in the active dimeric state^62^. Analogous to the role of the corresponding residue in Src-family kinases^64^, we see that Trp342 is also important for stabilizing the inactive, C-helix-out conformation of CRAF. Phosphorylation or mutation of the adjacent NtA motif apparently releases Trp342 from its inhibitory position. Phosphorylation of the NtA motif could be expected to promote kinase domain dimerization simply by destabilizing the inactive conformation, thereby lowering the energy barrier to dimerization, irrespective of a potential effect via direct binding interactions of the phosphorylated motif.

In part due to its role in KRAS-driven cancers, CRAF is a prominent target for development of anti-cancer therapeutics^48^. Because the kinase activity of CRAF is not essential for its role in oncogenesis, simply inhibiting CRAF is not expected to be a viable approach^45,46^. Targeted degradation of CRAF has been suggested as one alternative^44,45^. Although CRAF kinase activity is dispensable, its ability to dimerize is required^45^. The autoinhibited state blocks dimerization and holds CRAF in an inactive state, but is relatively unstable. Thus, small molecules that stabilize the fully autoinhibited state, perhaps by cementing interactions of the CRD with the kinase domain and/or 14-3-3 dimer, would be an attractive route to blocking both the catalytic activity and scaffolding/dimerization functions of CRAF.

## Methods

### Protein expression and purification

#### Full-length CRAF^SSYY^/MEK1/14-3-3, CRAF^SSDD^/MEK1/14-3-3, and BRAF/MEK1/14-3-3 complexes

Recombinant baculovirus expressing full-length CRAF with the Q156R and D587E mutations and either the wild-type (^338^SSYY^341^) NtA motif or the Y340D/Y341D (SSDD) mutant was prepared using the pAC8 baculoviral transfer vector, which contains an N-terminal His_6_-tag and a C-terminal Strep-tag. The baculovirus for the full-length wild-type BRAF was prepared as previously described^18^. For protein expression using the baculovirus system and *Sf9* cells, liter-scale cultures of *Sf9* cells were infected with high-titer baculoviral stocks expressing the RAF construct of interest at 0.5% of the final culture volume. Co-expression with MEK1^SASA^ was accomplished by co-infection with a second recombinant baculovirus expressing full-length MEK1 with the S218A/S222A mutation and an N-terminal His_6_-tag at an equal volume. After 72 hours post-infection, cells were harvested by centrifugation at 1500 g, then lysed in buffer containing 50 mM Tris, pH 7.4, 150 mM NaCl, 2 mM MgCl_2_, 0.5 mM TCEP, 15 mM imidazole, 20 µM ATPγS, 1 µM GDC-0623, and protease inhibitor cocktail (Thermo Fisher Scientific) using sonication. The lysates were subjected to ultracentrifugation at 40,000 rpm (186,010 g at the max radius) for 1.5 hours and applied to a HisTrap HP column (Cytiva) equilibrated with Buffer A (50 mM Tris, pH 7.5, 150 mM NaCl, 2 mM MgCl_2_, 0.5 mM TCEP, 2 µM ATPγS, 1 µM GDC-0623). After washing with Buffer A supplemented with 30 mM imidazole, the proteins were eluted with Buffer B (50 mM Tris pH 8.0, 150 mM NaCl, 2 mM MgCl_2_, 0.5 mM TCEP, 300 mM imidazole, 2 µM ATPγS, 1 µM GDC-0623). The eluted proteins were then applied to a StrepTrap HP column (Cytiva) equilibrated with Buffer A. Following a wash with Buffer A, the bound proteins were eluted with Buffer A supplemented with 10 mM desthiobiotin at pH 7.5 and subsequently injected onto a HiLoad 16/600 Superose 6 pg column (Cytiva) equilibrated with Buffer A. The proteins from the main peak were concentrated and applied to a Superdex200 Increase 10/300 GL column (Cytiva), also equilibrated with Buffer A. The final protein solution was concentrated to approximately 1 mg/ml using an Amicon Ultra concentrator (50 kDa MWCO, Millipore). Purified proteins were analyzed by SDS-PAGE, which confirmed that the co-expressed RAF and MEK1 proteins were co-eluted with stoichiometric amounts of endogenous insect 14-3-3ε and 14-3-3ζ, forming the desired CRAF/MEK1/14-3-3 or BRAF/MEK1/14-3-3 complex.

#### CRAF^ΔN·SSYY^/MEK1/14-3-3, CRAF^ΔN·SSDD^/MEK1/14-3-3, and BRAF^ΔN^/MEK1^SASA^/14-3-3

Expression and purification of the N-terminally truncated active RAF dimers was performed as previously described^56^. Briefly, N-terminally truncated wild-type CRAF (CRAF^ΔN·SSYY^: residues 308 – 648) or Y340D/Y341D mutant CRAF (CRAF^ΔN·SSDD^: residues 308 – 648), or N-terminal truncated wild-type BRAF (BRAF^ΔN^: residues 419 – 766) were co-expressed with full-length MEK1. CRAF^ΔN·SSDD^ and BRAF^ΔN^ were co-expressed with mutant MEK1 (S218A/S222A) and CRAF^ΔN·SSYY^ was co-expressed with wild-type MEK1. Sf9 cells were harvested by centrifugation and lysed by sonication in Ni^2+^-binding buffer (pH 8.0, 50 mM Tris, 150 mM NaCl, 10 mM MgCl_2_, 1 mM TCEP, 1 μM AMP-PNP, and protease inhibitor cocktail (Thermo Fisher Scientific). The lysate was applied to Ni^2+^-NTA agarose beads (Qiagen) equilibrated with Ni^2+^-binding buffer, followed by washing with Ni^2+^-binding buffer supplemented with 30 mM imidazole. Bound proteins were eluted with Ni^2+^-binding buffer supplemented with 250 mM imidazole. The eluted fractions were concentrated by Amicon Ultra concentrator (30 Kda MWCO, Millipore) and injected onto a Superdex 200 Increase 10/300 column (Cytiva). Purified proteins were analyzed by SDS-PAGE, which confirmed that the co-expressed RAF and MEK1 proteins were co-eluted with stoichiometric amounts of endogenous insect 14-3-3ε and 14-3-3ζ, forming the desired dimeric CRAF/MEK1/14-3-3 or BRAF/MEK1/14-3-3 complexes.

#### *SHOC2-PP1C-MRAS* complex

The *SHOC2-PP1C-MRAS* phosphatase complex was expressed and purified as previously described^78^. Briefly, recombinant baculoviruses expressing full-length SHOC2, MRAS (residues 1–178), and full-length PP1C fused to a His_6_-tag and maltose binding protein (MBP) were individually prepared and co-infected into liter-scale cultures of sf9 cells. A recombinant baculovirus expressing full-length SUGT1, a chaperone protein, was also co-infected to enhance the production yield and quality of the SHOC2-PP1C-MRAS complex. Cells were harvested 72 hours post-infection and lysed using sonication in buffer A (20 mM HEPES, pH 7.5, 300 mM NaCl, 1 mM TCEP, and 1 µM GMPPNP) supplemented with 10 µM GMPPNP and protease inhibitor cocktail (Thermo Fisher Scientific). The lysates were clarified by ultracentrifugation at 40,000 rpm (186,010 g at the max radius) for 1.5 hours and loaded onto a HisTrap HP column (Cytiva). After washing with buffer A supplemented with 40 mM imidazole, bound proteins were eluted using a linear gradient of buffer B (20 mM HEPES, pH 7.5, 300 mM NaCl, 1 mM TCEP, 500 mM imidazole, and 1 µM GMPPNP). The eluted proteins were subsequently buffer-exchanged to buffer A using HiPrep Desalting columns packed with Sephadex G-25 resin (Cytiva) and treated with TEV protease at a 1:50 ratio at 4 °C overnight. His-tagged TEV protease and cleaved His-MBP were retained on a HisTrap HP column (Cytiva), while the cleaved proteins were eluted using buffer A supplemented with 40 mM imidazole. The eluted fractions were concentrated by Amicon Ultra concentrator (50 kDa MWCO, Millipore) and injected onto a Superdex 200 Increase 10/300 column (Cytiva) equilibrated with buffer C (20 mM HEPES pH 7.5, 150 mM NaCl, 1 mM TCEP, and 1 µM GMPPNP). SDS-PAGE analysis confirmed the formation of the SHOC2-PP1C-MRAS complex in a stoichiometric ratio.

### Cryo-EM grid preparation and data acquisition

To prepare grids for cryo-EM, CRAF^SSYY^/MEK1^SASA^/14-3-3 or CRAF^SSDD^ /MEK1^SASA^/14-3-3 complex at a concentration of 0.4 mg/ml was prepared in buffer containing 50 mM Tris, pH 7.5, 150 mM NaCl, 2 mM MgCl_2_, 0.5 mM TCEP, 10 µM ATPγS, and 2 µM GDC-0623. The protein complexes were applied to holey gold grids (UltrAuFoil R 0.6/1.0, 300 mesh), which had been glow-discharged for 2 min at 20 mA using GloQube Plus (Quorum). Grids were blotted for 2–3 s, post-blotted for an additional 2 s using LEICA EM GP (Leica Microsystems) at 11 °C and 95% relative humidity, and then immediately plunged into liquid ethane.

The data collection was conducted on a Titan Krios (Thermo Fisher Scientific) at 300 keV equipped with a Falcon4i direct electron detector and Selectris Energy Filter operated using EPU. Approximately 50 frames per movie were collected at a magnification of 165,000x (corresponding to 0.73 Å per pixel) with a total exposure dose of ~50 electrons and a defocus range of 0.8 to 1.8 μm. For the kinase domain open monomer CRAF^SSYY^/MEK1^SASA^/14-3-3 complex, a total of 9,198 micrographs were collected on one grid. For the fully autoinhibited CRAF^SSYY^/MEK1^SASA^/14-3-3 complex, a total of 11,510 micrographs were collected on one grid. For the CRAF^SSDD^ MEK1^SASA^/14-3-3 complex, a total of 13,349 micrographs were collected on one grid, with 6060 of these acquired at a 30° tilt to increase observed orientation. Details of the data collection and dataset parameters are summarized in Supplementary Tables 1 and 2.

### Cryo-EM data processing and model building

For the CRAF^SSYY^/MEK1^SASA^/14-3-3 complex, 9198 movies were processed using Patch motion correction and Patch CTF estimation in CryoSPARC^79^. Initial particle picking with crYOLO^80^ yielded 1,320,979 particles, which were reduced to 905,495 through iterative 2D classification. Micrographs with CTF resolutions poorer than 4.8 Å were excluded, resulting in 6634 retained micrographs. New particles were picked using a Topaz^81^ model trained on the previous particles, yielding 1,213,559 particles, which were reduced to 967,637 particles through iterative 2D classification. To remove junk particles and distinguish between different conformations, ab initio reconstruction and heterogeneous refinement were carried out, specifying six 3D classes. This approach yielded three distinct classes representing different RAF complex configurations (177,653 particles for the autoinhibited complex, 258,542 particles for the kinase domain open monomer complex, and 176,231 particles for the open monomer complex). Each of the three classes was further refined individually via homogeneous refinement followed by non-uniform refinement. Particle orientations were well-distributed for the reconstruction of the kinase domain open monomer complex, but the autoinhibited complex and the open monomer complex reconstructions exhibited significant preferred orientations. To refine the kinase domain open monomer complex, multiple rounds of heterogeneous refinement using decoy volumes were conducted to remove residual junk particles. Following reference-based motion correction of the remaining particles, a final homogeneous refinement followed by non-uniform refinement yielded a map at 2.9 Å (unmasked). The overall scheme for the data processing is summarized in Supplementary Fig. 3.

To improve the map of the autoinhibited CRAF^SSYY^/MEK1^SASA^/14-3-3 complex, 11,510 movies were collected using a new grid and processed using a similar strategy as described above. Initial particle picking with crYOLO^80^ yielded 1,810,081 particles, which were reduced to 1,717,003 through 2D classification. New particles were picked with a Topaz^81^ model trained on the previous particles, yielding 1,749,602 particles, which were reduced to 1,742,278 particles through 2D classification. Ab initio reconstruction and heterogeneous refinement were performed with eight 3D classes, resulting in three distinct classes corresponding to the previously observed RAF complex configurations. Particle orientations were well-distributed for the autoinhibited complex and kinase domain open monomer complex, whereas the open monomer complex still exhibited substantial preferred orientations. To further refine the autoinhibited complex, multiple rounds of heterogeneous refinement using decoy volumes were used to remove residual junk particles. Following reference-based motion correction of the remaining particles, a final homogeneous refinement followed by non-uniform refinement yielded a map at 3.4 Å (unmasked). The overall scheme for the data processing is summarized in Supplementary Fig. 4.

For the CRAF^SSDD^/MEK1^SASA^/14-3-3 complex, 7289 movies were processed using Patch motion correction and Patch CTF estimation in CryoSPARC^79^. Particles were picked using a Topaz^81^ model trained on manually selected particles, yielding 2,814,493 particles. After removing junk particles through iterative 2D classification, 2,397,630 particles were used for ab initio reconstruction with five 3D classes. Particles of three 3D classes corresponding to the kinase monomer complex were further refined through additional rounds of 2D classification and ab initio reconstruction. A subset of 451,072 particles from the best 3D class was refined using homogenous refinement and non-uniform refinement. However, the resulting map still exhibited preferred orientation issues. To address this, template-based particle picking was carried out using the intermediate map, which yielded 2,647,126 particles. Extensive particle sorting was then conducted through iterative 2D classification, heterogeneous refinement with decoy volumes, and rebalance orientation in CryoSPARC^79^. The remaining 175,813 particles were subjected to reference-based motion correction, followed by homogenous refinement and non-uniform refinement, resulting in a final map at 3.3 Å (unmasked). The overall scheme for the data processing is summarized in Supplementary Fig. 5.

In this dataset, maps for both the autoinhibited complex and the open monomer complex were reconstructed. However, the number of particles corresponding to the autoinhibited complex was insufficient, and the open monomer complex exhibited severe preferred orientation. In order to mitigate the preferred orientation of particles in the open monomer state, 6060 additional movies were collected from the same grid at a 30° tilt. These tilted movies were processed using Patch motion correction and Patch CTF estimation in CryoSPARC^79^. Particle picking with crYOLO^80^ yielded 1,170,212 particles. Although data quality was reduced due in part to increased ice thickness, 101,829 particles for the open monomer complex were successfully isolated through 2D classification. These particles were subjected to ab initio reconstruction and heterogeneous refinement, resulting in the intermediate map with 76,924 particles. Using this map, template-based particle picking was performed, resulting in 2,223,388 particles. Extensive particle sorting was then carried out through iterative 2D classification and heterogeneous refinement with decoy volumes. The remaining 131,440 particles were subjected to reference-based motion correction, followed by homogenous refinement and non-uniform refinement, resulting in a final map at 3.9 Å (unmasked). The overall scheme for the data processing is summarized in Supplementary Fig. 6.

Local resolution estimates for all reconstructions, calculated in RELION^82^, are color-coded in the corresponding cryo-EM maps. Gold-standard Fourier shell correlation (GSFSC) curves and conical FSC curves assessing directional resolution are shown in Supplementary Fig. 7.

Atomic models were fit into cryo-EM maps using ChimeraX^83^ and were further refined with PHENIX^84^ and Coot^85^. PDB entry 9AY7 was used as an initial model for the CRAF^SSYY^ and MEK1^SASA^ kinase domains in the kinase domain open monomer, which was manually rebuilt and refined in the corresponding cryo-EM map. This refined CRAF^SSYY^/MEK1 model was used as an initial model for the autoinhibited CRAF^SSYY^/MEK1/14-3-3 complex, together with the 14-3-3 portion of PDB entry 6NYB and an Alphafold3-generated model for the CRD, pSer259, and pSer621 segments. The refined CRAF^SSYY^/MEK1 model was also used as an initial model for the CRAF^SSDD^ and MEK1^SASA^ kinase domains in the kinase domain open monomer, which was manually refined in the corresponding cryo-EM map. The refined CRAF^SSYY^/MEK1 mode,l together with the 14-3-3 portion of PDB entry 6NYB was used as an initial model for the CRAF^SSDD^/MEK1/14-3-3 open monomer, with reference to an Alphafold3 model for fitting of the pSer621 segment. Final refinement statistics for the CRAF^SSYY^/MEK1^SASA^/14-3-3 and CRAF^SSDD^/MEK1^SASA^/14-3-3 complex structures are presented in Supplementary Tables 1 and 2, respectively.

### RAF kinase activity assay

A time-resolved FRET (TR-FRET) assay was performed to measure RAF complex kinase activity using a modified HTRF KinEASE assay kit (Cisbio) essentially as previously described^56^. Briefly, RAF complexes were prepared at concentrations ranging from 100 nM to 49 pM through 2-fold serial dilutions in kinase buffer supplemented with 25 mM MgCl_2_ and 0.25 mM TCEP, along with 250 µM biotinylated-MEK1 as the substrate. The reactions were initiated by adding 200 µM ATP and incubated for 40 min at room temperature. The reactions were then quenched using detection buffer, supplemented with an anti-phospho MEK1/2 antibody coupled to Eu^3+^ as the FRET donor and XL665-streptavidin conjugate as the FRET acceptor. Following a 30-minute development, FRET signal ratios were measured at 665 and 620 nm using a PHERAstar microplate reader (BML).

### RAF dephosphorylation assay

RAF complexes at a concentration of 200 nM were incubated with the SHOC2-PP1C-MRAS complex at concentrations of 400 nM, 200 nM, 100 nM, 50 nM, and 25 nM for 90 min at 37 °C in a buffer containing 20 mM HEPES pH 7.5, 150 mM NaCl, 2 mM MgCl_2_, 2 mM MnCl_2_, 1 mM TCEP, 0.02% Brij 35. Reaction products were analyzed by Western blot using phospho-specific antibody for CRAF phosphorylated at Ser259 or BRAF phosphorylated at Ser365 (Cell Signaling Technology, #9421). For a loading control, the membranes were stripped using stripping buffer (Thermo Scientific) and re-probed with antibodies for against CRAF or BRAF (Cell Signaling Technology, #53745 for CRAF and #14814 for BRAF).

Following dephosphorylation, RAF complex activity was assessed using two approaches: the first measuring phosphorylation of MEK1 in an HTRF assay (Cisbio) and the second measuring phosphorylation of ERK in a cascade assay via Western blot. For the HTRF assay, reaction products were diluted to 20 nM RAF complex in kinase buffer supplemented with 25 mM MgCl_2_, 0.25 mM TCEP, and 1 mM Na_3_VO_4_, along with 250 µM biotinylated-MEK1 as the substrate. Reactions were initiated by adding 200 µM ATP and incubated at the indicated time points. At each time point, the reactions were quenched using detection buffer, supplemented with an anti-phospho MEK1/2 antibody coupled to Eu^3+^ as the FRET donor and XL665-streptavidin conjugate as the FRET acceptor. Following a 30-minute development, FRET signal ratios were measured at 665 and 620 nm using a PHERAstar microplate reader.

In the ERK cascade assay, reaction products were diluted to 5 nM RAF complex in a buffer containing 20 mM HEPES pH 7.5, 150 mM NaCl, 2 mM MgCl_2_, 1 mM TCEP, and 1 mM Na_3_VO_4_, and incubated with 10 nM MEK1 and 4 μM ERK2 in the presence of 1 mM ATP. At the indicated time points, reactions were quenched by adding 2x SDS sample buffer followed by heat inactivation for 5 min at 85°C. Assay results were analyzed by Western blot using a phospho-ERK1/2 antibody (Cell Signaling Technology, #45899). For a loading control, the membranes were stripped using stripping buffer (Thermo Scientific) and re-probed with an anti-ERK1/2 antibody (Cell Signaling Technology, #9102).

### Mass spectrometry analysis

Proteins were denatured in 8 M urea in HEPES pH 8. Disulfide bonds were reduced in 5 mM TCEP for 30 minutes and alkylated with 10 mM iodoacetamide for 45 minutes in the dark. Reactions were quenched with DTT. Urea was diluted to 1 M with 50 mM HEPES, pH 8, prior to digestion with trypsin (1:50 enzyme: protein) for 16 hours at 32 °C. Digests were acidified and desalted over C18 resin (SOLAμ, Thermo Fisher Scientific).

Digested peptides were analyzed on an Orbitrap Eclipse mass spectrometer coupled to an Ultimate 3000 RSLCnano (Thermo Fisher Scientific). Peptides were separated over a 50-cm C18 column (ES903, Thermo Fisher Scientific) with a 70-min gradient of 6-30% acetonitrile in 0.1% formic acid and electrosprayed (1.9 kV, 300 °C) with an EasySpray ion source. Precursor ion scans (375-1,325 m/z) were obtained in the Orbitrap (120,000 resolution, profile). Data-dependent MS^2^ scans (*n* = 2 in 15 s, exclusion duration = 30 s) were acquired in the Orbitrap following HCD fragmentation (35% NCE, 0.7 m/z isolation, 30,000 resolution).

Raw data were searched against a sequence database containing the engineered protein sequences and common contaminants using SEQUEST in Proteome Discoverer 2.4, permitting a mass tolerance of ±10 ppm, 2 missed cleavages by trypsin, static carbamidomethylation of Cys, and the following variable modifications: oxidation of Met, phosphorylation of Ser/Thr/Tyr. Peptide spectral matches were validated using a target/decoy approach (1% FDR). Spectra of identified phosphopeptides were manually validated to confirm accurate site localization. Precursor ions were quantified with the Minora Feature Detector, Feature Mapper, and Precursor Ion Quantifier nodes. Phosphorylation site occupancy was estimated from the ratio of phosphopeptide abundance to the summed abundance of all species, phosphorylated and unphosphorylated, containing each site of interest.

### Size-exclusion chromatography with multiangle light scattering

The CRAF^SSDD^/MEK1/14-3-3 complex was applied to a Superdex 200 Increase 10/300 column (Cytiva) in the buffer containing 50 mM Tris-HCl pH 7.5, 150 mM NaCl, 2 mM MgCl_2_, 0.5 mM TCEP, 2 μM ATPγS, and 1 μM GDC-0623. In-line multi-angle light scattering analysis was performed with an OptiLab rEX refractive index detector followed by a miniDAWN TREOS light scattering detector, and data were analyzed with ASTRA (Wyatt Technology).

### Reporting summary

Further information on research design is available in the Nature Portfolio Reporting Summary linked to this article.

## Supplementary information

Supplementary Information

Reporting Summary

Transparent Peer Review file

## Source data

Source Data

## Data Availability

The cryo-EM maps of the CRAF^SSYY^/MEK1^SASA^/14-3-3 complex in the autoinhibited conformation and kinase domain open monomer conformation were deposited to the EM Data Bank (https://www.ebi.ac.uk/emdb/) under accession codes EMD-48397 and EMD-48399, respectively. Cryo-EM maps for the CRAF^SSDD^/MEK1^SASA^/14-3-3 complex in the open monomer conformation and kinase domain open monomer conformation were deposited to the EM Data Bank under accession codes EMD-48401 and EMD-48402, respectively. Atomic models for the CRAF^SSYY^/MEK1^SASA^/14-3-3 complex in the autoinhibited conformation and kinase domain open monomer conformation were deposited to the Protein Data Bank (PDB) and are available at www.rcsb.org under accession codes 9MMP and 9MMQ. Atomic models for the CRAF^SSDD^/MEK1^SASA^/14-3-3 complex in the open monomer conformation and kinase domain open monomer conformations were deposited to the PDB under accession codes 9MMR. and 9MMS, respectively. Detailed information for all maps and models generated in this work is provided in Supplementary Tables 1 and 2. Source data including mass spectrometry analysis and unprocessed western blots are provided with this paper. Source data are provided with this paper.

## References

[1] Lavoie, H. & Therrien, M. Regulation of RAF protein kinases in ERK signalling. *Nat. Rev. Mol. Cell Biol.* **16**, 281–298 (2015). 25907612 10.1038/nrm3979

[2] Terrell, E. M. & Morrison, D. K. Ras-Mediated activation of the Raf family kinases. *Cold Spring Harb. Perspect. Med.* **9**, a033746 (2019). 10.1101/cshperspect.a033746 PMC6311149 29358316

[3] Jeon, H., Tkacik, E. & Eck, M. J. Signaling from RAS to RAF: The molecules and their mechanisms. *Annu Rev. Biochem* **93**, 289–316 (2024). 38316136 10.1146/annurev-biochem-052521-040754

[4] Simanshu, D. K., Nissley, D. V. & McCormick, F. RAS proteins and their regulators in human disease. *Cell* **170**, 17–33 (2017). 28666118 10.1016/j.cell.2017.06.009 PMC5555610

[5] Desideri, E., Cavallo, A. L. & Baccarini, M. Alike but different: RAF paralogs and their signaling outputs. *Cell* **161**, 967–970 (2015). 26000477 10.1016/j.cell.2015.04.045

[6] Marais, R., Light, Y., Paterson, H. F., Mason, C. S. & Marshall, C. J. Differential regulation of Raf-1, A-Raf, and B-Raf by oncogenic ras and tyrosine kinases. *J. Biol. Chem.* **272**, 4378–4383 (1997). 9020159 10.1074/jbc.272.7.4378

[7] Emuss, V., Garnett, M., Mason, C. & Marais, R. Mutations of C-RAF are rare in human cancer because C-RAF has a low basal kinase activity compared with B-RAF. *Cancer Res.* **65**, 9719–9726 (2005). 16266992 10.1158/0008-5472.CAN-05-1683

[8] Sanchez-Vega, F. et al. Oncogenic signaling pathways in the Cancer Genome Atlas. *Cell* **173**, 321–337.e10 (2018). 29625050 10.1016/j.cell.2018.03.035 PMC6070353

[9] Ghosh, S. et al. The cysteine-rich region of Raf-1 kinase contains zinc, translocates to liposomes, and is adjacent to a segment that binds GTP-Ras. *J. Biol. Chem.* **269**, 10000–10007 (1994). 8144497

[10] Tran, T. H. et al. KRAS interaction with RAF1 RAS-binding domain and cysteine-rich domain provides insights into RAS-mediated RAF activation. *Nat. Commun.* **12**, 1176 (2021). 33608534 10.1038/s41467-021-21422-x PMC7895934

[11] Haling, J. R. et al. Structure of the BRAF-MEK complex reveals a kinase activity independent role for BRAF in MAPK signaling. *Cancer Cell* **26**, 402–413 (2014). 25155755 10.1016/j.ccr.2014.07.007

[12] Diedrich, B. et al. Discrete cytosolic macromolecular BRAF complexes exhibit distinct activities and composition. *EMBO J.* **36**, 646–663 (2017). 28093501 10.15252/embj.201694732 PMC5331759

[13] Fantl, W. J. et al. Activation of Raf-1 by 14-3-3 proteins. *Nature* **371**, 612–614 (1994). 7935795 10.1038/371612a0

[14] Freed, E., Symons, M., Macdonald, S. G., McCormick, F. & Ruggieri, R. Binding of 14-3-3 proteins to the protein kinase Raf and effects on its activation. *Science* **265**, 1713–1716 (1994). 8085158 10.1126/science.8085158

[15] Fu, H. et al. Interaction of the protein kinase Raf-1 with 14-3-3 proteins. *Science* **266**, 126–129 (1994). 7939632 10.1126/science.7939632

[16] Yaffe, M. B. et al. The structural basis for 14-3-3:phosphopeptide binding specificity. *Cell* **91**, 961–971 (1997). 9428519 10.1016/s0092-8674(00)80487-0

[17] Muslin, A. J., Tanner, J. W., Allen, P. M. & Shaw, A. S. Interaction of 14-3-3 with signaling proteins is mediated by the recognition of phosphoserine. *Cell* **84**, 889–897 (1996). 8601312 10.1016/s0092-8674(00)81067-3

[18] Park, E. et al. Architecture of autoinhibited and active BRAF-MEK1-14-3-3 complexes. *Nature* **575**, 545–550 (2019). 31581174 10.1038/s41586-019-1660-y PMC7014971

[19] Martinez Fiesco, J. A., Durrant, D. E., Morrison, D. K. & Zhang, P. Structural insights into the BRAF monomer-to-dimer transition mediated by RAS binding. *Nat. Commun.* **13**, 486 (2022). 35078985 10.1038/s41467-022-28084-3 PMC8789793

[20] Zhang, X. F. et al. Normal and oncogenic p21ras proteins bind to the amino-terminal regulatory domain of c-Raf-1. *Nature* **364**, 308–313 (1993). 8332187 10.1038/364308a0

[21] Vojtek, A. B., Hollenberg, S. M. & Cooper, J. A. Mammalian Ras interacts directly with the serine/threonine kinase Raf. *Cell* **74**, 205–214 (1993). 8334704 10.1016/0092-8674(93)90307-c

[22] Fang, Z. et al. Multivalent assembly of KRAS with the RAS-binding and cysteine-rich domains of CRAF on the membrane. *Proc. Natl. Acad. Sci. USA* **117**, 12101–12108 (2020). 32414921 10.1073/pnas.1914076117 PMC7275734

[23] Hekman, M. et al. Associations of B- and C-Raf with cholesterol, phosphatidylserine, and lipid second messengers: preferential binding of Raf to artificial lipid rafts. *J. Biol. Chem.* **277**, 24090–24102 (2002). 11953426 10.1074/jbc.M200576200

[24] Kondo, Y. et al. Cryo-EM structure of a dimeric B-Raf:14-3-3 complex reveals asymmetry in the active sites of B-Raf kinases. *Science* **366**, 109–115 (2019). 31604311 10.1126/science.aay0543 PMC7007921

[25] Liau, N. P. D. et al. Negative regulation of RAF kinase activity by ATP is overcome by 14-3-3-induced dimerization. *Nat. Struct. Mol. Biol.* **27**, 134–141 (2020). 31988522 10.1038/s41594-019-0365-0

[26] Rodriguez-Viciana, P., Oses-Prieto, J., Burlingame, A., Fried, M. & McCormick, F. A phosphatase holoenzyme comprised of Shoc2/Sur8 and the catalytic subunit of PP1 functions as an M-Ras effector to modulate Raf activity. *Mol. Cell* **22**, 217–230 (2006). 16630891 10.1016/j.molcel.2006.03.027

[27] Young, L. C. et al. SHOC2-MRAS-PP1 complex positively regulates RAF activity and contributes to Noonan syndrome pathogenesis. *Proc. Natl. Acad. Sci. USA* **115**, E10576–E10585 (2018). 30348783 10.1073/pnas.1720352115 PMC6233131

[28] Hauseman, Z. J. et al. Structure of the MRAS-SHOC2-PP1C phosphatase complex. *Nature* **609**, 416–423 (2022). 35830882 10.1038/s41586-022-05086-1 PMC9452295

[29] Kwon, J. J. et al. Structure-function analysis of the SHOC2-MRAS-PP1C holophosphatase complex. *Nature* **609**, 408–415 (2022). 35831509 10.1038/s41586-022-04928-2 PMC9694338

[30] Liau, N. P. D. et al. Structural basis for SHOC2 modulation of RAS signalling. *Nature* **609**, 400–407 (2022). 35768504 10.1038/s41586-022-04838-3 PMC9452301

[31] Bonsor, D. A. et al. Structure of the SHOC2-MRAS-PP1C complex provides insights into RAF activation and Noonan syndrome. *Nat. Struct. Mol. Biol.* **29**, 966–977 (2022). 36175670 10.1038/s41594-022-00841-4 PMC10365013

[32] Park, E. et al. Cryo-EM structure of a RAS/RAF recruitment complex. *Nat. Commun.* **14**, 4580 (2023). 37516774 10.1038/s41467-023-40299-6 PMC10387098

[33] Fabian, J. R., Daar, I. O. & Morrison, D. K. Critical tyrosine residues regulate the enzymatic and biological activity of Raf-1 kinase. *Mol. Cell Biol.* **13**, 7170–7179 (1993). 7692235 10.1128/mcb.13.11.7170-7179.1993 PMC364778

[34] Mason, C. S. et al. Serine and tyrosine phosphorylations cooperate in Raf-1, but not B-Raf activation. *EMBO J.* **18**, 2137–2148 (1999). 10205168 10.1093/emboj/18.8.2137 PMC1171298

[35] Diaz, B. et al. Phosphorylation of Raf-1 serine 338-serine 339 is an essential regulatory event for Ras-dependent activation and biological signaling. *Mol. Cell Biol.* **17**, 4509–4516 (1997). 9234708 10.1128/mcb.17.8.4509 PMC232304

[36] Tran, N. H. & Frost, J. A. Phosphorylation of Raf-1 by p21-activated kinase 1 and Src regulates Raf-1 autoinhibition. *J. Biol. Chem.* **278**, 11221–11226 (2003). 12551923 10.1074/jbc.M210318200

[37] King, A. J., Wireman, R. S., Hamilton, M. & Marshall, M. S. Phosphorylation site specificity of the Pak-mediated regulation of Raf-1 and cooperativity with Src. *FEBS Lett.* **497**, 6–14 (2001). 11376654 10.1016/s0014-5793(01)02425-5

[38] Zang, M., Hayne, C. & Luo, Z. Interaction between active Pak1 and Raf-1 is necessary for phosphorylation and activation of Raf-1. *J. Biol. Chem.* **277**, 4395–4405 (2002). 11733498 10.1074/jbc.M110000200

[39] Kolch, W. et al. Protein kinase C alpha activates RAF-1 by direct phosphorylation. *Nature* **364**, 249–252 (1993). 8321321 10.1038/364249a0

[40] Karreth, F. A., Frese, K. K., DeNicola, G. M., Baccarini, M. & Tuveson, D. A. C-Raf is required for the initiation of lung cancer by K-Ras(G12D). *Cancer Discov.* **1**, 128–136 (2011). 22043453 10.1158/2159-8290.CD-10-0044 PMC3203527

[41] McCormick, F. c-Raf in KRas mutant cancers: a moving target. *Cancer Cell* **33**, 158–159 (2018). 29438690 10.1016/j.ccell.2018.01.017 PMC6464632

[42] Blasco, R. B. et al. c-Raf, but not B-Raf, is essential for development of K-Ras oncogene-driven non-small cell lung carcinoma. *Cancer Cell* **19**, 652–663 (2011). 21514245 10.1016/j.ccr.2011.04.002 PMC4854330

[43] Sanclemente, M. et al. c-RAF ablation induces regression of advanced Kras/Trp53 mutant Lung Adenocarcinomas by a mechanism independent of MAPK Signaling. *Cancer Cell* **33**, 217–228.e4 (2018). 29395869 10.1016/j.ccell.2017.12.014

[44] Blasco, M. T. et al. Complete regression of advanced pancreatic ductal adenocarcinomas upon combined inhibition of EGFR and C-RAF. *Cancer Cell* **35**, 573–587.e6 (2019). 30975481 10.1016/j.ccell.2019.03.002 PMC10132447

[45] Venkatanarayan, A. et al. CRAF dimerization with ARAF regulates KRAS-driven tumor growth. *Cell Rep.* **38**, 110351 (2022). 35139374 10.1016/j.celrep.2022.110351

[46] Sanclemente, M. et al. RAF1 kinase activity is dispensable for KRAS/p53 mutant lung tumor progression. *Cancer Cell* **39**, 294–296 (2021). 33513349 10.1016/j.ccell.2021.01.008

[47] Heidorn, S. J. et al. Kinase-dead BRAF and oncogenic RAS cooperate to drive tumor progression through CRAF. *Cell* **140**, 209–221 (2010). 20141835 10.1016/j.cell.2009.12.040 PMC2872605

[48] Riaud, M. et al. The role of CRAF in cancer progression: from molecular mechanisms to precision therapies. *Nat. Rev. Cancer* **24**, 105–122 (2024). 38195917 10.1038/s41568-023-00650-x

[49] Pandit, B. et al. Gain-of-function RAF1 mutations cause Noonan and LEOPARD syndromes with hypertrophic cardiomyopathy. *Nat. Genet.* **39**, 1007–1012 (2007). 17603483 10.1038/ng2073

[50] Kobayashi, T. et al. Molecular and clinical analysis of RAF1 in Noonan syndrome and related disorders: dephosphorylation of serine 259 as the essential mechanism for mutant activation. *Hum. Mutat.* **31**, 284–294 (2010). 20052757 10.1002/humu.21187

[51] Tartaglia, M., Aoki, Y. & Gelb, B. D. The molecular genetics of RASopathies: An update on novel disease genes and new disorders. *Am. J. Med. Genet. C. Semin Med. Genet.* **190**, 425–439 (2022). 36394128 10.1002/ajmg.c.32012 PMC10100036

[52] Holderfield, M., Deuker, M. M., McCormick, F. & McMahon, M. Targeting RAF kinases for cancer therapy: BRAF-mutated melanoma and beyond. *Nat. Rev. Cancer* **14**, 455–467 (2014). 24957944 10.1038/nrc3760 PMC4250230

[53] Dedden, D. et al. Cryo-EM Structures of CRAF(2)/14-3-3(2) and CRAF(2)/14-3-3(2)/MEK1(2) Complexes. *J. Mol. Biol.* **436**, 168483 (2024). 38331211 10.1016/j.jmb.2024.168483

[54] Ryan, M. B. et al. The Pan-RAF-MEK nondegrading molecular glue NST-628 Is a potent and brain-penetrant inhibitor of the RAS-MAPK pathway with activity across diverse RAS- and RAF-driven cancers. *Cancer Discov.* **14**, 1190–1205 (2024). 38588399 10.1158/2159-8290.CD-24-0139 PMC11215411

[55] Takahashi, M., Li, Y., Dillon, T. J., Kariya, Y. & Stork, P. J. S. Phosphorylation of the C-Raf N region promotes Raf dimerization. *Mol. Cell Biol*. **37**, e00132-17 (2017). 10.1128/MCB.00132-17 PMC5599720 28694330

[56] Tkacik, E. et al. Structure and RAF family kinase isoform selectivity of type II RAF inhibitors tovorafenib and naporafenib. *J. Biol. Chem.* **299**, 104634 (2023). 36963492 10.1016/j.jbc.2023.104634 PMC10149214

[57] Abramson, J. et al. Accurate structure prediction of biomolecular interactions with AlphaFold 3. *Nature* **630**, 493–500 (2024). 38718835 10.1038/s41586-024-07487-w PMC11168924

[58] Freeman, A. K., Ritt, D. A. & Morrison, D. K. Effects of Raf dimerization and its inhibition on normal and disease-associated Raf signaling. *Mol. Cell* **49**, 751–758 (2013). 23352452 10.1016/j.molcel.2012.12.018 PMC3582845

[59] Daub, M. et al. The RafC1 cysteine-rich domain contains multiple distinct regulatory epitopes which control Ras-dependent Raf activation. *Mol. Cell Biol.* **18**, 6698–6710 (1998). 9774683 10.1128/mcb.18.11.6698 PMC109253

[60] Gonzalez-Del Pino, G. L. et al. Allosteric MEK inhibitors act on BRAF/MEK complexes to block MEK activation. *Proc. Natl. Acad. Sci. USA* **118**, e2107207118 (2021). 34470822 10.1073/pnas.2107207118 PMC8433572

[61] Rajakulendran, T., Sahmi, M., Lefrancois, M., Sicheri, F. & Therrien, M. A dimerization-dependent mechanism drives RAF catalytic activation. *Nature* **461**, 542–545 (2009). 19727074 10.1038/nature08314

[62] Hu, J. et al. Allosteric activation of functionally asymmetric RAF kinase dimers. *Cell* **154**, 1036–1046 (2013). 23993095 10.1016/j.cell.2013.07.046 PMC3844432

[63] Jambrina, P. G. et al. Phosphorylation of RAF kinase dimers drives conformational changes that facilitate transactivation. *Angew. Chem. Int Ed. Engl.* **55**, 983–986 (2016). 26644280 10.1002/anie.201509272 PMC4736688

[64] LaFevre-Bernt, M. et al. Intramolecular regulatory interactions in the Src family kinase Hck probed by mutagenesis of a conserved tryptophan residue. *J. Biol. Chem.* **273**, 32129–32134 (1998). 9822689 10.1074/jbc.273.48.32129

[65] Weber, C. K., Slupsky, J. R., Kalmes, H. A. & Rapp, U. R. Active Ras induces heterodimerization of cRaf and BRaf. *Cancer Res.* **61**, 3595–3598 (2001). 11325826

[66] Garnett, M. J., Rana, S., Paterson, H., Barford, D. & Marais, R. Wild-type and mutant B-RAF activate C-RAF through distinct mechanisms involving heterodimerization. *Mol. Cell* **20**, 963–969 (2005). 16364920 10.1016/j.molcel.2005.10.022

[67] Rushworth, L. K., Hindley, A. D., O’Neill, E. & Kolch, W. Regulation and role of Raf-1/B-Raf heterodimerization. *Mol. Cell Biol.* **26**, 2262–2272 (2006). 16508002 10.1128/MCB.26.6.2262-2272.2006 PMC1430271

[68] Ritt, D. A., Monson, D. M., Specht, S. I. & Morrison, D. K. Impact of feedback phosphorylation and Raf heterodimerization on normal and mutant B-Raf signaling. *Mol. Cell Biol.* **30**, 806–819 (2010). 19933846 10.1128/MCB.00569-09 PMC2812223

[69] Schulte, T. W., Blagosklonny, M. V., Ingui, C. & Neckers, L. Disruption of the Raf-1-Hsp90 molecular complex results in destabilization of Raf-1 and loss of Raf-1-Ras association. *J. Biol. Chem.* **270**, 24585–24588 (1995). 7592678 10.1074/jbc.270.41.24585

[70] Grammatikakis, N., Lin, J. H., Grammatikakis, A., Tsichlis, P. N. & Cochran, B. H. p50(cdc37) acting in concert with Hsp90 is required for Raf-1 function. *Mol. Cell Biol.* **19**, 1661–1672 (1999). 10022854 10.1128/mcb.19.3.1661 PMC83960

[71] Taipale, M. et al. Quantitative analysis of HSP90-client interactions reveals principles of substrate recognition. *Cell* **150**, 987–1001 (2012). 22939624 10.1016/j.cell.2012.06.047 PMC3894786

[72] Garcia-Alonso, S. et al. Structure of the RAF1-HSP90-CDC37 complex reveals the basis of RAF1 regulation. *Mol. Cell* **82**, 3438–3452.e8 (2022). 36055235 10.1016/j.molcel.2022.08.012

[73] Spencer-Smith, R. et al. RASopathy mutations provide functional insight into the BRAF cysteine-rich domain and reveal the importance of autoinhibition in BRAF regulation. *Mol. Cell* **82**, 4262–4276.e5 (2022). 36347258 10.1016/j.molcel.2022.10.016 PMC9677513

[74] Wang, P., Laster, K., Jia, X., Dong, Z. & Liu, K. Targeting CRAF kinase in anti-cancer therapy: progress and opportunities. *Mol. Cancer* **22**, 208 (2023). 38111008 10.1186/s12943-023-01903-x PMC10726672

[75] Williams, N. G., Roberts, T. M. & Li, P. Both p21ras and pp60v-src are required, but neither alone is sufficient, to activate the Raf-1 kinase. *Proc. Natl. Acad. Sci. USA* **89**, 2922–2926 (1992). 1372995 10.1073/pnas.89.7.2922 PMC48775

[76] Stokoe, D. & McCormick, F. Activation of c-Raf-1 by Ras and Src through different mechanisms: activation in vivo and in vitro. *EMBO J.* **16**, 2384–2396 (1997). 9171352 10.1093/emboj/16.9.2384 PMC1169839

[77] Cutler, R. E. Jr., Stephens, R. M., Saracino, M. R. & Morrison, D. K. Autoregulation of the Raf-1 serine/threonine kinase. *Proc. Natl. Acad. Sci. USA* **95**, 9214–9219 (1998). 9689060 10.1073/pnas.95.16.9214 PMC21318

[78] Snead, K., Wall, V., Ambrose, H., Esposito, D. & Drew, M. Polycistronic baculovirus expression of SUGT1 enables high-yield production of recombinant leucine-rich repeat proteins and protein complexes. *Protein Expr. Purif.* **193**, 106061 (2022). 35131438 10.1016/j.pep.2022.106061 PMC8881745

[79] Punjani, A., Rubinstein, J. L., Fleet, D. J. & Brubaker, M. A. cryoSPARC: algorithms for rapid unsupervised cryo-EM structure determination. *Nat. Methods* **14**, 290–296 (2017). 28165473 10.1038/nmeth.4169

[80] Wagner, T. et al. SPHIRE-crYOLO is a fast and accurate fully automated particle picker for cryo-EM. *Commun. Biol.* **2**, 218 (2019). 31240256 10.1038/s42003-019-0437-z PMC6584505

[81] Bepler, T. et al. Positive-unlabeled convolutional neural networks for particle picking in cryo-electron micrographs. *Nat. Methods* **16**, 1153–1160 (2019). 31591578 10.1038/s41592-019-0575-8 PMC6858545

[82] Kucukelbir, A., Sigworth, F. J. & Tagare, H. D. Quantifying the local resolution of cryo-EM density maps. *Nat. Methods* **11**, 63–65 (2014). 24213166 10.1038/nmeth.2727 PMC3903095

[83] Meng, E. C. et al. UCSF ChimeraX: Tools for structure building and analysis. *Protein Sci.* **32**, e4792 (2023). 37774136 10.1002/pro.4792 PMC10588335

[84] Adams, P. D. et al. PHENIX: a comprehensive Python-based system for macromolecular structure solution. *Acta Crystallogr D. Biol. Crystallogr* **66**, 213–221 (2010). 20124702 10.1107/S0907444909052925 PMC2815670

[85] Emsley, P., Lohkamp, B., Scott, W. G. & Cowtan, K. Features and development of Coot. *Acta Crystallogr D. Biol. Crystallogr* **66**, 486–501 (2010). 20383002 10.1107/S0907444910007493 PMC2852313

