# Supplementary material for: Cryo-EM structures of CRAF/MEK1/14-3-3 complexes in autoinhibited and open-monomer states reveal features of RAF regulation
Source: Nat Commun. 2025 Sep 1;16:8150. doi: 10.1038/s41467-025-63227-2 (PMC12402067; doi:10.1038/s41467-025-63227-2)
Supplement: Supplementary file 1 — Supplementary Information [file 41467_2025_63227_MOESM1_ESM.pdf]

Supplementary Information

Cryo-EM structures of CRAF/MEK1/14-3-3 complexes in autoinhibited and open-monomer states reveal features of RAF regulation.

Dong Man Jang[1,2], Kayla Boxer[1], Byung Hak Ha[1,2], Emre Tkacik[1,2,3], Talya Levitz[1], Shaun Rawson[2], Rebecca J. Metivier[1], Anna Schmoker[1], Hyesung Jeon[1,2], and Michael J. Eck[1,2*]

[1]Department of Cancer Biology, Dana-Farber Cancer Institute, Boston, MA 02215, USA.
[2]Department of Biological Chemistry and Molecular Pharmacology, Harvard Medical School, Boston, MA 02115, USA.
[3]Systems, Synthetic, and Quantitative Biology PhD Program, Harvard Medical School, Boston, Massachusetts, USA.

*Corresponding Author:
Michael J. Eck
e-mail: Michael_Eck@dfci.harvard.edu

Supplementary Information

- Supplementary Tables 1 – 3
- Supplementary Figures 1 – 13

Supplementary Table 1 | Cryo-EM data collection, refinement and validation statistics for the CRAF^{SSYY}/MEK1/14-3-3 complex.

**Data collection and processing**		
Magnification	165,000 x	165,000 x
Voltage (kV)	300	300
Electron exposure (e/Å^2)	53.67	51.34
Defocus range (μm)	− 0.8 – −1.8	− 0.8 – −1.8
Pixel size	0.73	0.73
Symmetry imposed	C1	C1
Number of micrographs	9,198	11,510
**Refinement**	**Kinase domain open monomer**	**Autoinhibited monomer**
	(PDB 9MMQ) (EMD-48399)	(PDB 9MMP) (EMD-48397)
Final particle images (no.)	168,600	128,302
Map resolution (Å)		
0.143 FSC threshold	2.9 (unmasked), 2.3 (masked)	3.4 (unmasked), 2.9 (masked)
Initial model used (PDB code)	9ay7	6nyb, AlphaFold, 9mmq
Model composition		
Chains	2	4
Non-hydrogen atoms	4461	8758
Protein residues	551	1085
Ligands	3	3
Metals	2	4
B factors (Å^2)		
Proteins (min/max/average)	57.23/163.38/92.57	30.00/255.52/130.36
Ligands (min/max/average)	63.92/139.62/83.88	101.39/275.58/153.93
R.m.s. deviations		
Bond length (Å)	0.005	0.005
Bond angles (°)	0.684	0.810
Validation		
MolProbity score	1.73	1.74
Clash score	10.11	10.66
Poor rotamers (%)	0.21	0.53
Ramachandran plot		
Favored (%)	96.70	96.80
Allowed (%)	3.30	3.20
Outliers (%)	0.0	0.00
Model vs. Data		
CC (mask)	0.81	0.80
CC (box)	0.74	0.81
CC (peaks)	0.69	0.67
CC (volume)	0.81	0.79
Mean CC for ligands	0.77	0.74

Supplementary Table 2 | Cryo-EM data collection, refinement and validation statistics for the CRAF^{SSDD}/MEK1/14-3-3 complex.

**Data collection and processing**		
Magnification	165,000 x	165,000 x
Voltage (kV)	300	300
Electron exposure (e/Å^2)	51.26	56.65
Defocus range (μm)	− 0.8 – −1.8	− 0.8 – −1.8
Pixel size	0.73	0.73
Symmetry imposed	C1	C1
Number of micrographs	7,289	6,060 (30° tilted)
**Refinement**	**Kinase domain open monomer**	**Open monomer**
	(PDB 9MMS) (EMD-48402)	(PDB 9MMR) (EMD-48401)
Final particle images (no.)	175,214	131,061
Map resolution (Å)		
0.143 FSC threshold	3.3 (unmasked), 2.8 (masked)	3.9 (unmasked), 3.3 (masked)
Initial model used (PDB code)	9mmq	6nyb, 9mmq
Model composition		
Chains	2	4
Non-hydrogen atoms	4410	8182
Protein residues	546	1020
Ligands	3	3
Metals	1	2
B factors (Å^2)		
Proteins (min/max/average)	96.33/270.15/152.98	127.18/298.68/183.83
Ligands (min/max/average)	113.27/213.11/139.38	160.01/228.36/187.73
R.m.s. deviations		
Bond length (Å)	0.004	0.004
Bond angles (°)	0.844	0.636
Validation		
MolProbity score	1.93	1.71
Clash score	20.98	12.58
Poor rotamers (%)	0.63	0.57
Ramachandran plot		
Favored (%)	97.41	97.52
Allowed (%)	2.59	2.48
Outliers (%)	0.0	0.00
Model vs. Data		
CC (mask)	0.69	0.74
CC (box)	0.75	0.81
CC (peaks)	0.58	0.62
CC (volume)	0.69	0.74
Mean CC for ligands	0.63	0.82

Supplementary Table 3 | Phosphorylation analysis of CRAF/MEK1/14-3-3 complexes by mass spectrometry.

		CRAF^{SSYY}/MEK1/14-3-3		CRAF^{SSDD}/MEK1/14-3-3		CRAF^{SSYY}/MEK1/14-3-3 + PAK1&Src1[1]	
**Protein**	**Site**	**% Phosphorylation**		**% Phosphorylation**		**% Phosphorylation**	
**CRAF**	S233 – T234[2]	2.1%	2.7%	3.7%	2.0%	9.1%	4.1%
	S259	97.3%	95.9%	86.3%	78.6%	98.2%	98.1%
	S285 – S301[3]	55.7%	47.2%	66.3%	60.4%	64.4%	66.8%
	S338	-	-	37.8%	42.6%	-	-
	S357	-	0.6%	56.3%	5.1%	0.4%	3.3%
	S497 – S499[2]	1.4%	1.3%	3.2%	14.6%	5.0%	-
	S621	98.0%	99.6%	100%[4]	94.0%	99.6%	98.3%
	S642	63.5%	90.0%	76.2%	18.7%	65.8%	81.2%

Two independent experiments show % phosphorylation at the respective sites.
- Not detected.
[1] Activated variants of PAK1 and Src1 were co-expressed with CRAF^{SSYY} and MEK1 in insect cells.
[2] Cannot distinguish between these two sites.
[3] Cannot distinguish among S285/S287/S289/S295/S296/S301.
[4] Unphosphorylated species not detected.

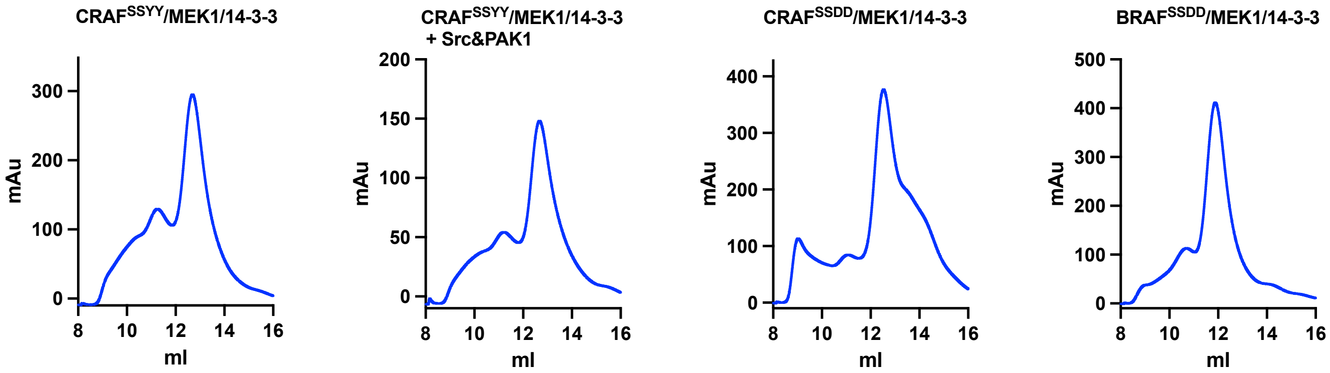

Supplementary Figure 1. Size exclusion chromatography profiles of full-length RAF complexes.

The indicated full-length RAF complexes were analyzed by size-exclusion chromatography on a Superdex 200 Increase 10/300 GL column after purification by serial Ni-NTA affinity chromatography, Strep-tag affinity chromatography, and size-exclusion chromatography on a HiLoad 16/600 Superose 6 pg column. The resulting UV_{280} absorbance trace is shown in blue. CRAF^{SSYY}/MEK1/14-3-3 + Src&PAK1 indicates co-expression of this complex with activated variants of PAK1 (PAK1 T423E) and c-Src (c-Src Y530F).

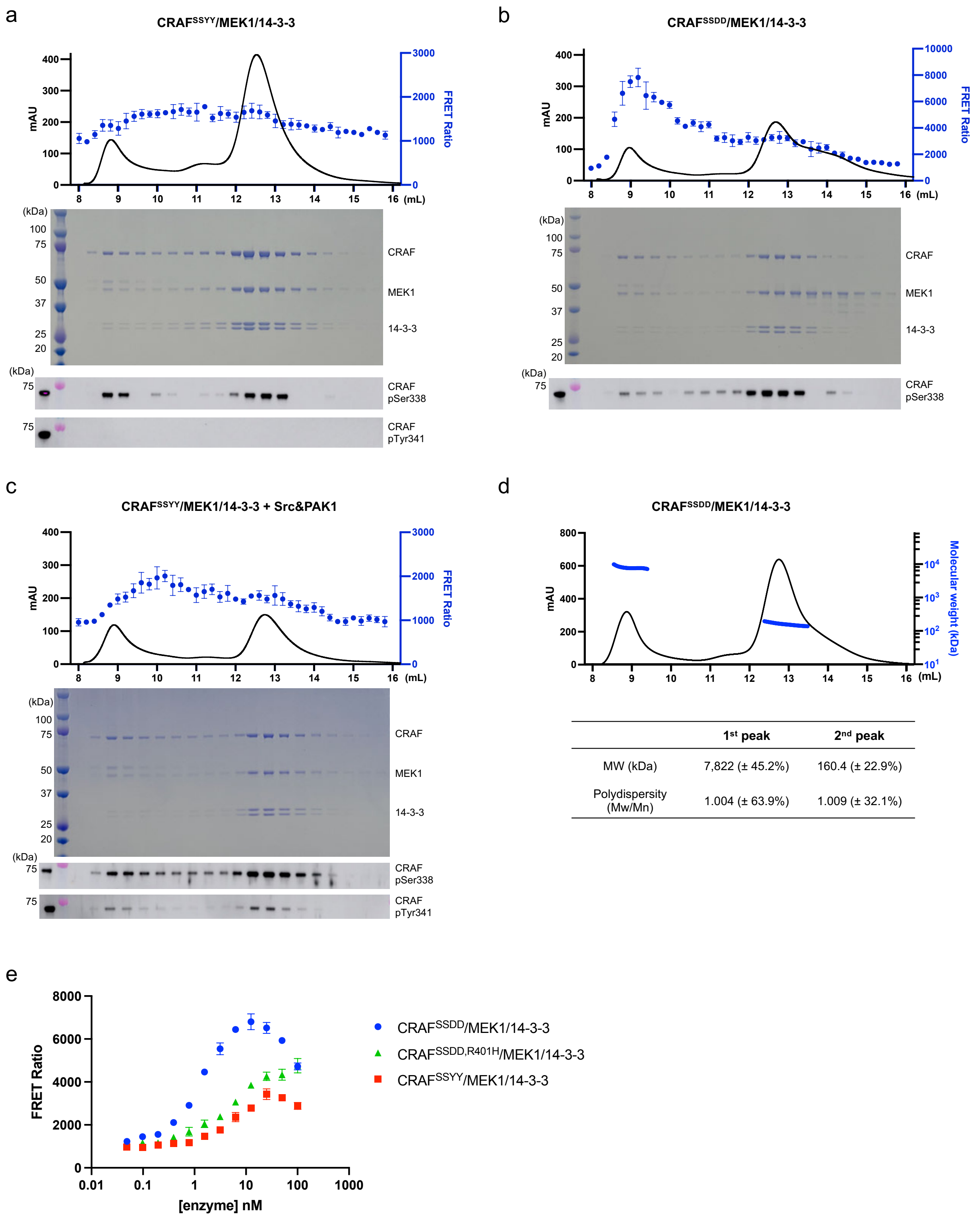

	1st peak	2nd peak
MW (kDa)	7,822 (± 45.2%)	160.4 (± 22.9%)
Polydispersity (Mw/Mn)	1.004 (± 63.9%)	1.009 (± 32.1%)

Supplementary Figure 2. Kinase activity and phosphorylation status of CRAF/MEK1/14-3-3 complexes followed by the size-exclusion chromatography.

The full-length CRAF^{SSYY}/MEK1/14-3-3 complex (**a**), the CRAF^{SSDD}/MEK1/14-3-3 complex (**b**), and the CRAF^{SSYY}/MEK1/14-3-3 complex co-expressed with Src and PAK1 (**c**) were subjected to size-exclusion chromatography (SEC) using a Superdex

200 Increase 10/300 GL column after initial purification by Ni-NTA and Strep-tag affinity chromatography. High-molecular-weight aggregates eluted at ~ 9 ml, as an initial Superose 6 SEC step was not applied. The resulting UV_{280} absorbance trace (black line) is shown in the top panel. The kinase activity of each fraction was measured using the TR-FRET-based assay (blue dots). Kinase activity is represented as the FRET ratio at 665/620 nm, with each point showing the mean ± SD of triplicate measurements. Note that the scale for the FRET ratio differs in the $CRAF^{SSDD}$/MEK1/14-3-3 complex. A Coomassie-stained SDS-PAGE gel for every other fraction is shown in the middle panel. The phosphorylation status of the NtA motif of each CRAF complex was assessed by western blot analysis with phospho-specific antibodies against pS338 (Cell Signaling Technology, #9427) and pY341 (Abcam, #ab59223) (bottom panels). An aliquot of the $CRAF^{SSYY}$/MEK1/14-3-3 complex co-expressed with c-Src Y530F and PAK1 T423E was run in the left-most lane as a positive control for the western blots. (**d**) Size-exclusion chromatography coupled with multi-angle light scattering (SEC-MALS) analysis of the $CRAF^{SSDD}$/MEK1/14-3-3 complex. The UV_{280} absorbance trace is shown as a black line, and the calculated molecular weight across the elution profile is indicated by blue dots. The estimated molecular weight and polydispersity index for each peak are summarized in the table below. The polydispersity index is calculated as the ratio of the weight-average molar mass (Mw) to the number-average molar mass (Mn). Note that the 1st peak, eluting at 8.9 ml, corresponds to high-molecular-weight aggregates and the 2nd peak at 12.8 ml corresponds to the monomeric CRAF/MEK1/14-3-3 complex. (**e**) Kinase activity of purified CRAF/MEK1/14-3-3 complexes measured using a TR-FRET based biochemical assay. SEC fractions corresponding to the monomeric complexes were used in the assay. Note that introduction of the dimer-disrupting R401H mutation in the CRAF kinase domain largely ablates the increased activity of the $CRAF^{SSDD}$ variant, indicating that $CRAF^{SSDD}$ remains dependent on dimerization for most of its activity. Considering that these complexes are monomeric as judged by SEC (panels a-d), we conclude that the increased activity of $CRAF^{SSDD}$ arises largely from transient dimerization.

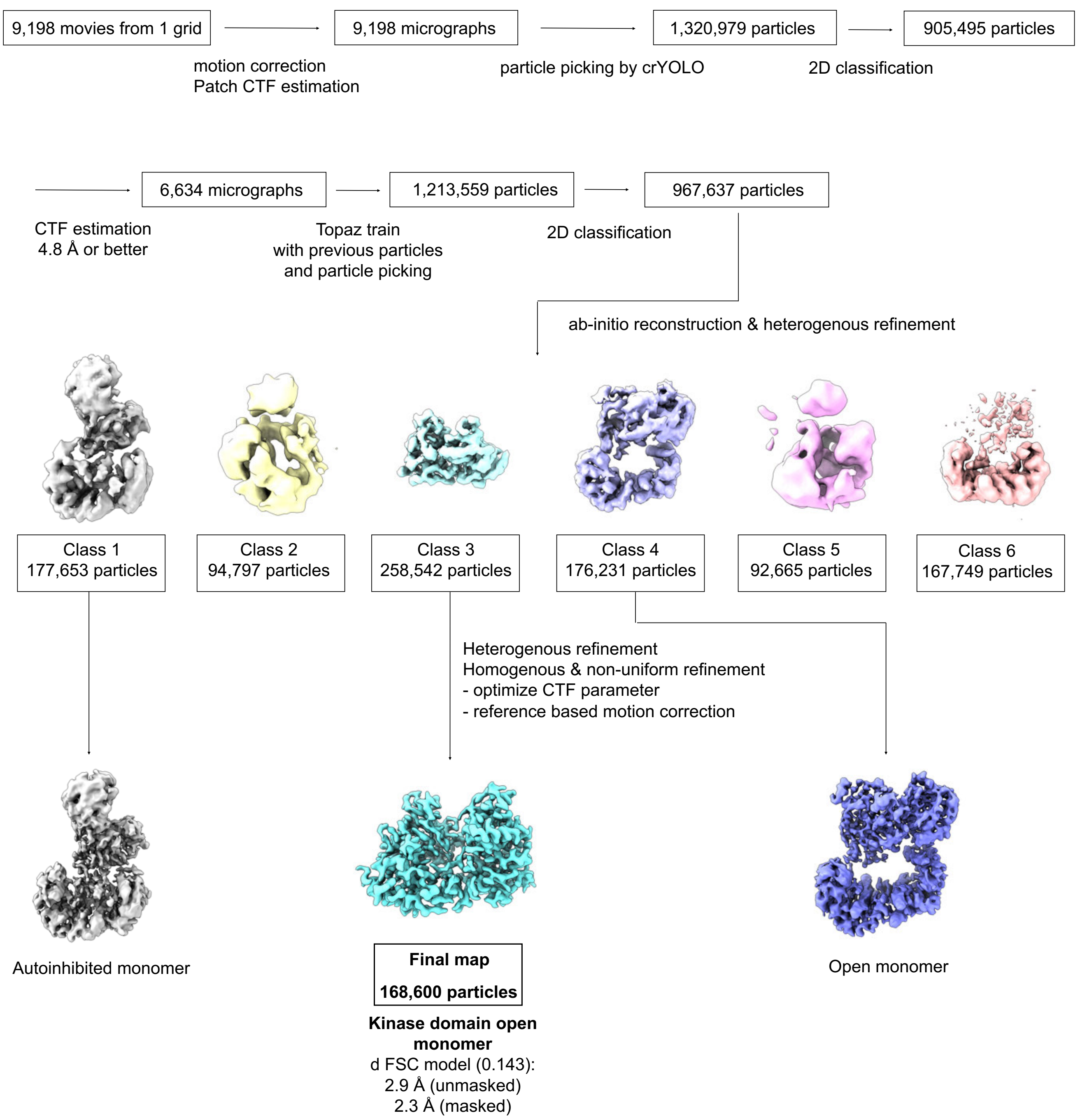

Supplementary Figure 3. Cryo-EM data processing workflow for the dataset 1: CRAF^{SSYY}/MEK1/14-3-3 in the kinase domain open monomer conformation.

Note that although both autoinhibited and open monomeric forms were present in the dataset, severe preferred orientation limited the map quality, and these states were therefore not pursued further.

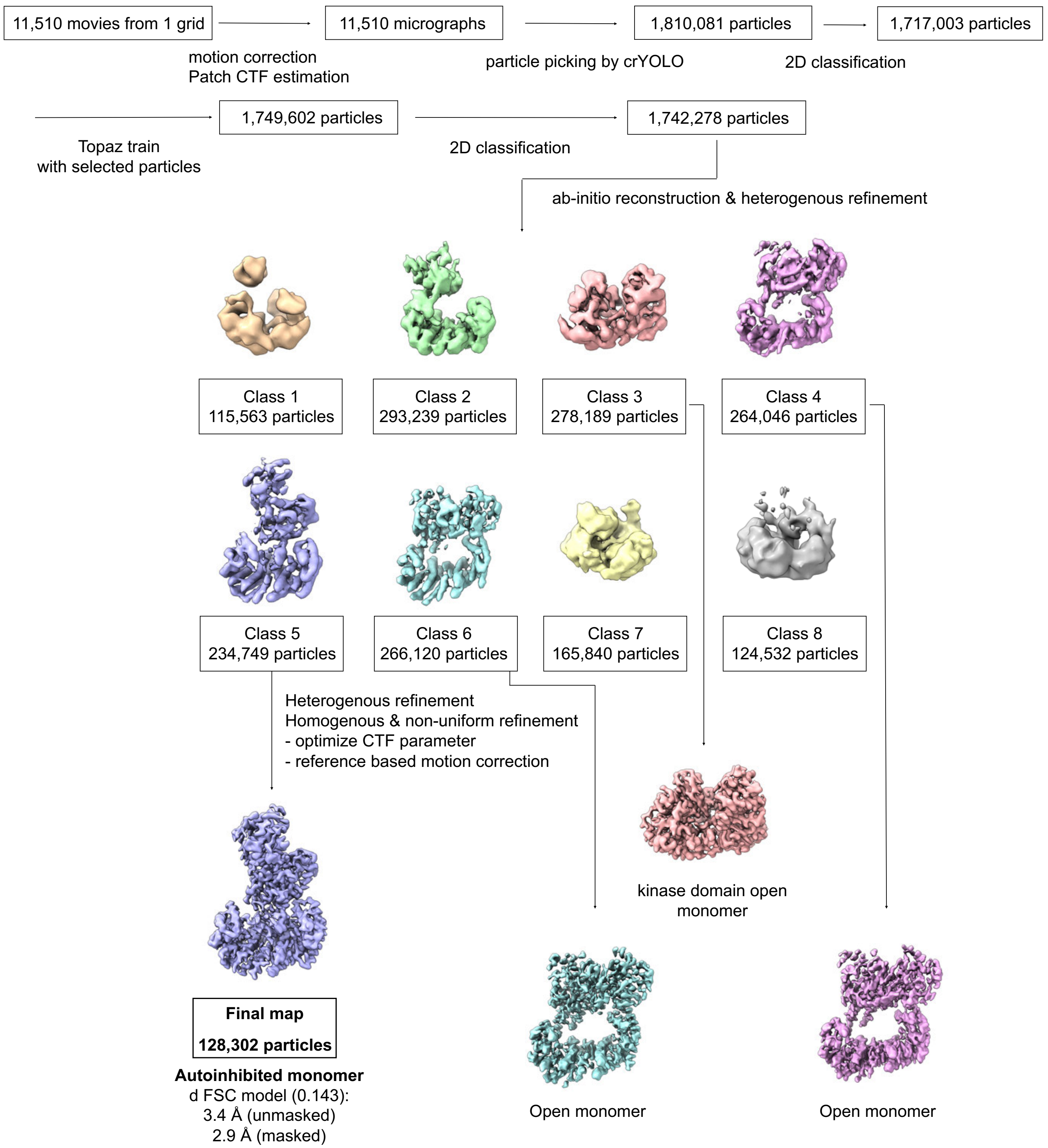

Supplementary Figure 4. Cryo-EM data processing workflow for the dataset 2: CRAF^{SSYY}/MEK1/14-3-3 in the autoinhibited conformation.

Note that although open monomeric form was present in the dataset, severe preferred orientation limited the map quality, and this state was therefore not pursued further.

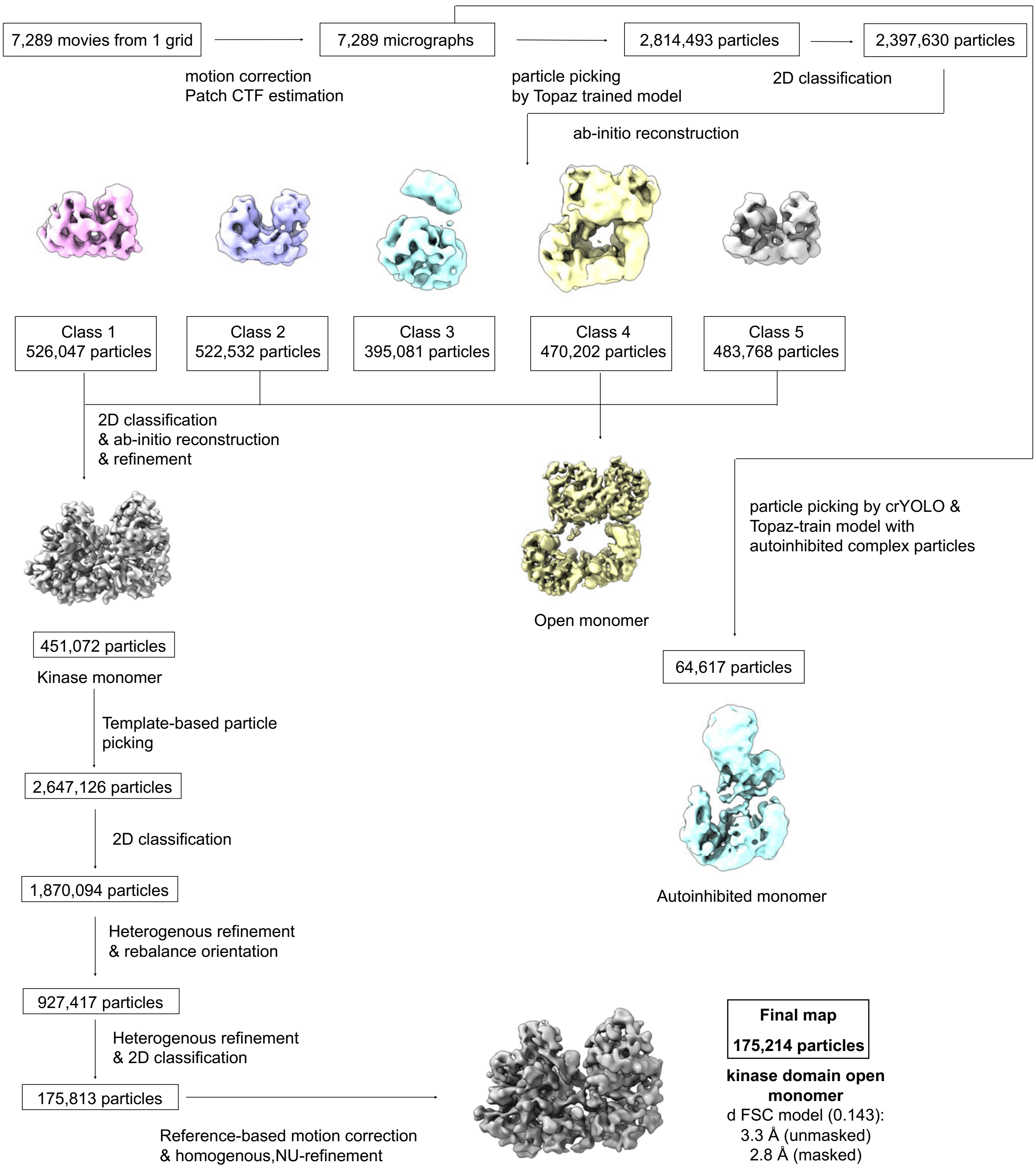

Supplementary Figure 5. Cryo-EM data processing workflow for the dataset 3: CRAF^{SSDD}/MEK1/14-3-3 in the kinase domain open monomer conformation.

Note that although autoinhibited and open monomeric forms were present in the dataset, severe preferred orientation limited the map quality, and these states were therefore not pursued further.

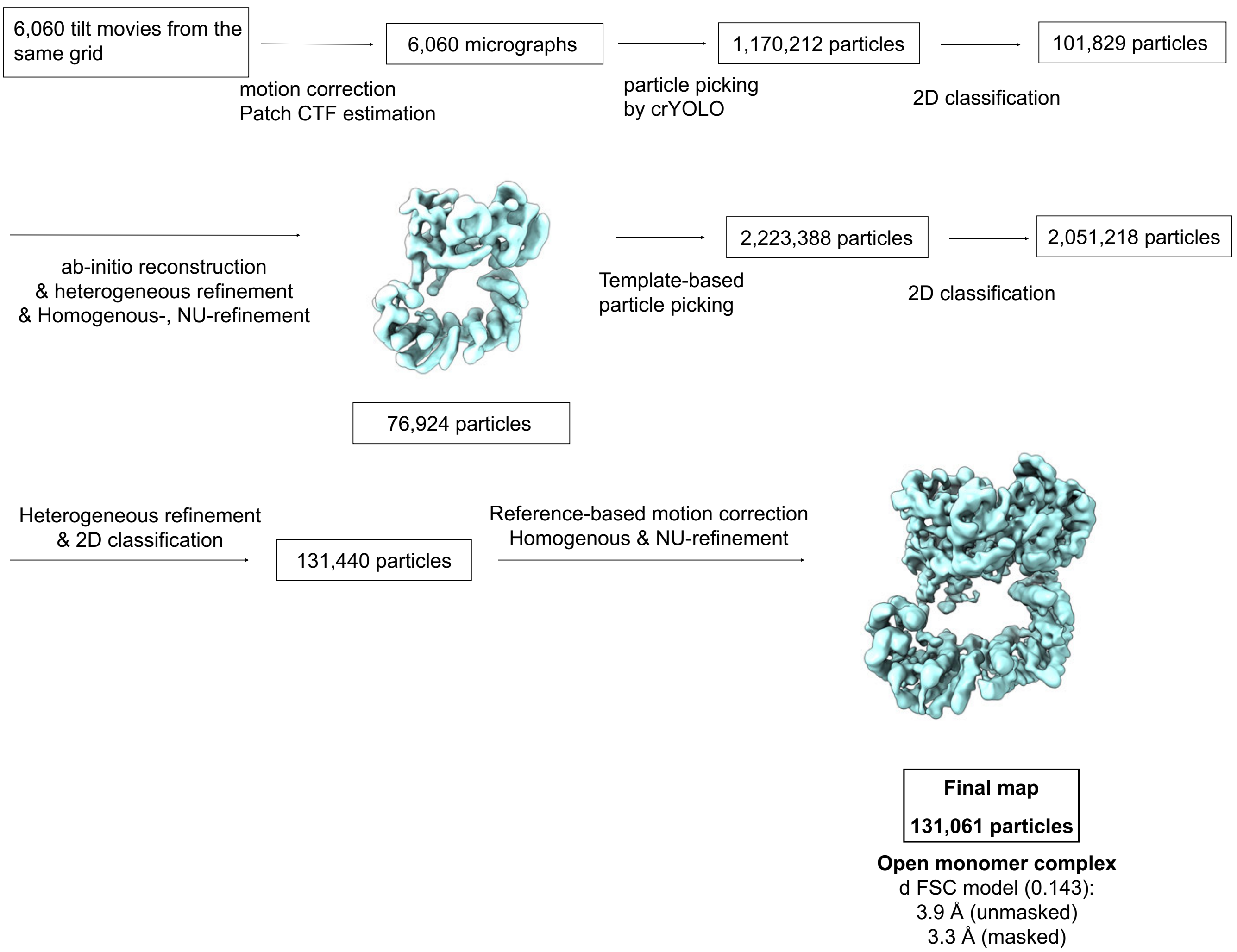

Supplementary Figure 6. Cryo-EM data processing workflow for the dataset 4: CRAF^{SSDD}/MEK1/14-3-3 in the open monomer conformation.

Note that the same grid with the dataset 3 was used for the tilt data collection.

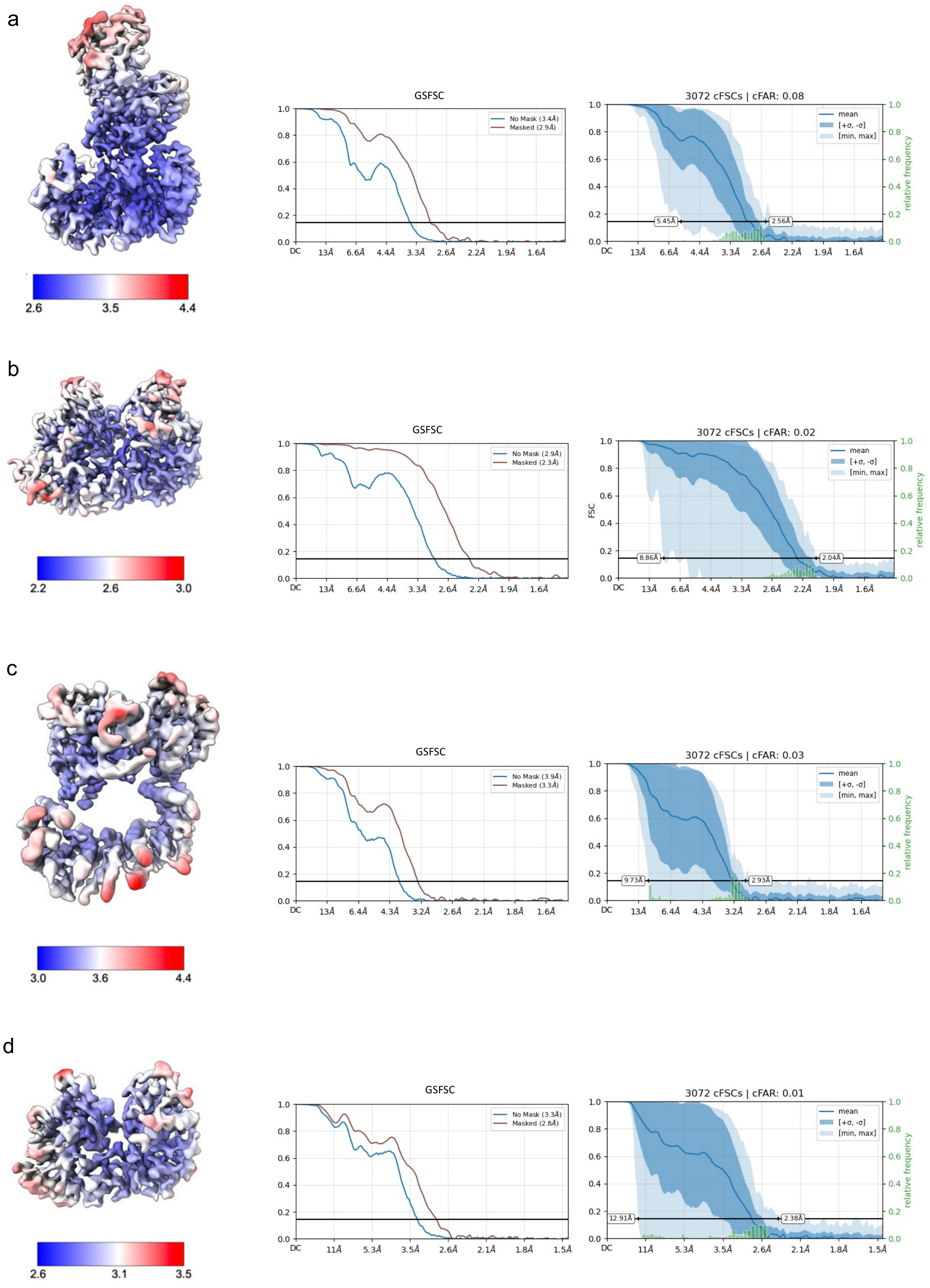

Supplementary Figure 7. Supporting information for cryo-EM structure determination.
Cryo-EM density maps colored by resolution for (**a**) CRAF^{SSYY}/MEK1/14-3-3 complex in the autoinhibited conformation, (**b**) CRAF^{SSYY}/MEK1 complex in the kinase domain open monomer conformation, (**c**) CRAF^{SSDD}/MEK1/14-3-3 complex in the open monomer conformation, and (**d**) CRAF^{SSDD}/MEK1 complex in the kinase domain open monomer conformation. Each map is shown with its corresponding gold-standard Fourier shell correlation (GSFSC) curves and conical Fourier shell correlation (cFSC) plots, as calculated in CryoSPARC.

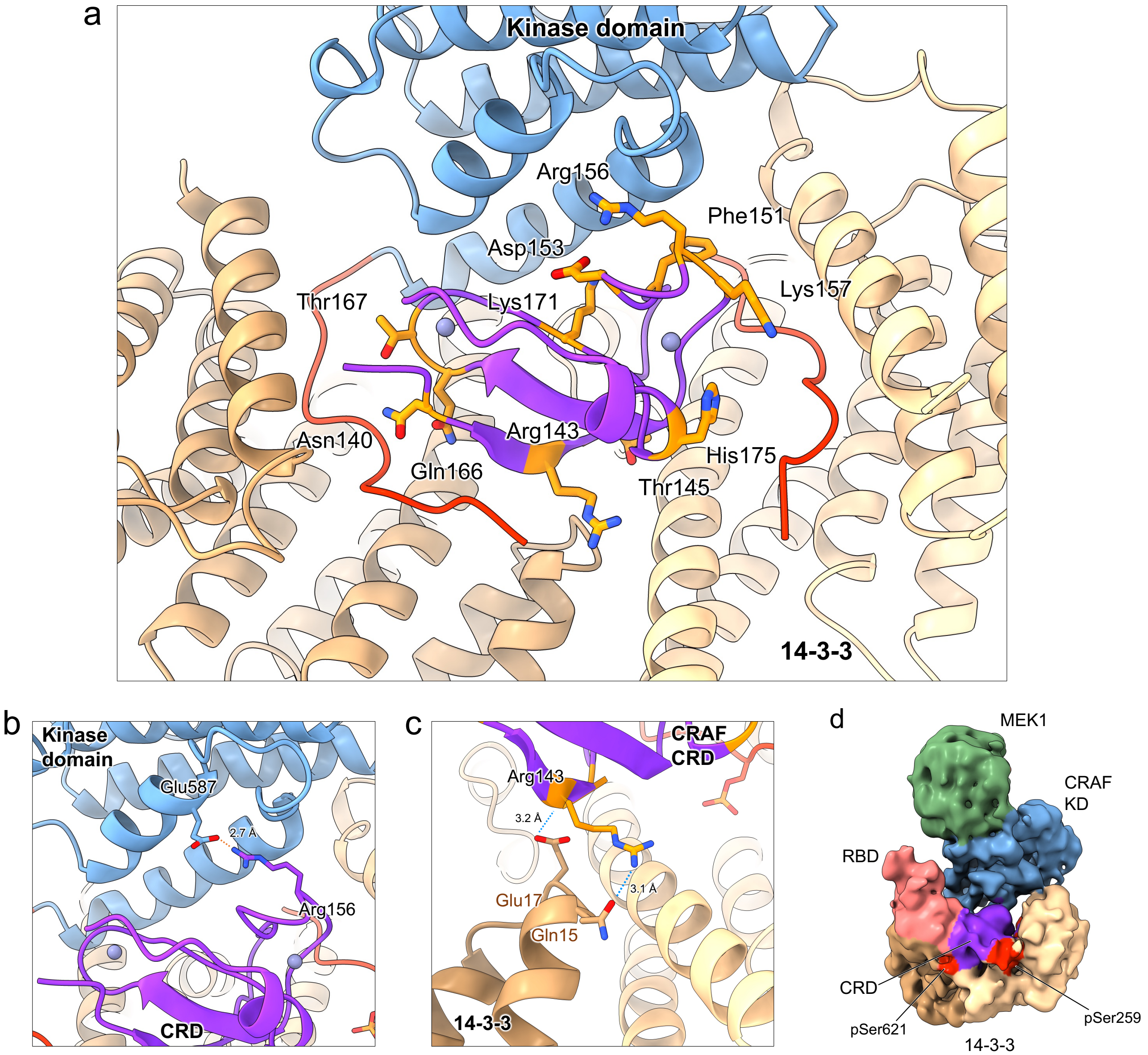

Supplementary Fig 8. Structural analysis of autoinhibited CRAF^{SSYY}/MEK1/14-3-3 complex.

(**a**) In the autoinhibited CRAF^{SSYY}/MEK1/14-3-3 complex, the CRAF kinase domain and CRD domain are colored in blue and purple, respectively. The 14-3-3 dimer is shown in tan and the phosphorylated pSer259 and pSer621 sites are shown in red. The residues on the CRD, involved in the prior mutagenesis study, are shown in stick representation colored in orange. (**b**) The mutant residues, Arg156 and Glu587, are shown in stick representation. (**c**) The residues at the interface of the CRAF CRD and 14-3-3 are displayed in stick representations. (**d**) The cryo-EM map of the autoinhibited CRAF^{SSYY}/MEK1/14-3-3 complex, low-pass filtered to 6 Å and contoured at a lower threshold, reveals weak density for the RBD, shown in salmon.

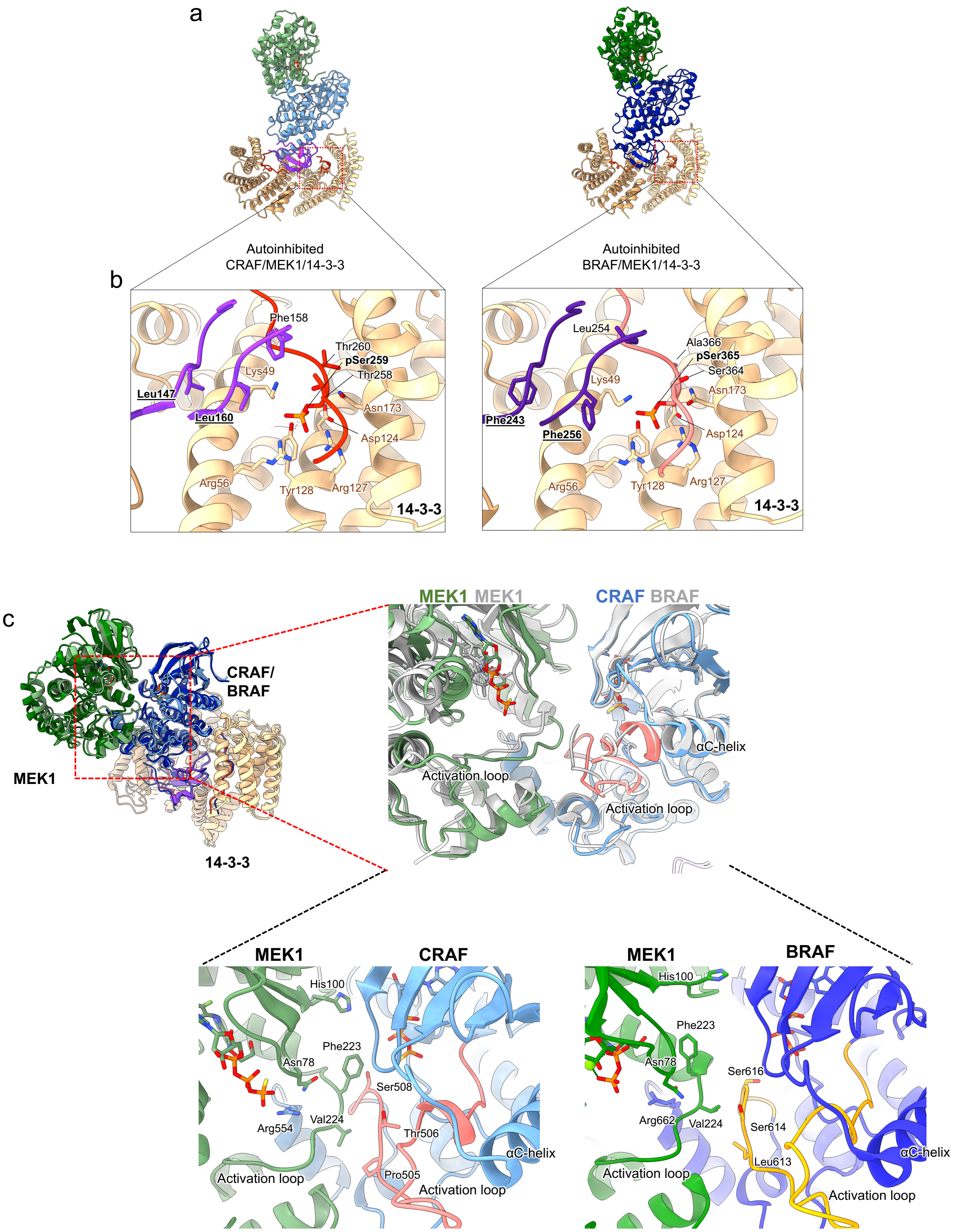

Supplementary Fig 9. Structural comparison of autoinhibited CRAF^{SSYY}/MEK1/14-3-3 and autoinhibited BRAF/MEK1/14-3-3 complexes.

(**a**) Overall structures of the autoinhibited CRAF^{SSYY}/MEK1/14-3-3 and autoinhibited BRAF/MEK1/14-3-3 (PDB ID: 6NYB) complexes. MEK1 is shown in green. The RAF kinase domain, CRD domain, and phosphorylated serine sites are colored in blue, purple, and red, respectively. The 14-3-3 dimer is depicted in tan. (**b**) Close-up views of the CRAF pSer259 and BRAF p365 binding sites. Residues in the interface are shown as stick representations. (**c**) Structural superimposition of the autoinhibited complexes of CRAF and BRAF based on RAF. Inset highlights the interface of RAF and MEK1. BRAF and its bound MEK1 are colored in grey, whereas CRAF is shown in blue and salmon, and MEK1 in green. The same RAF–MEK1 interface is shown separately below for each complex.

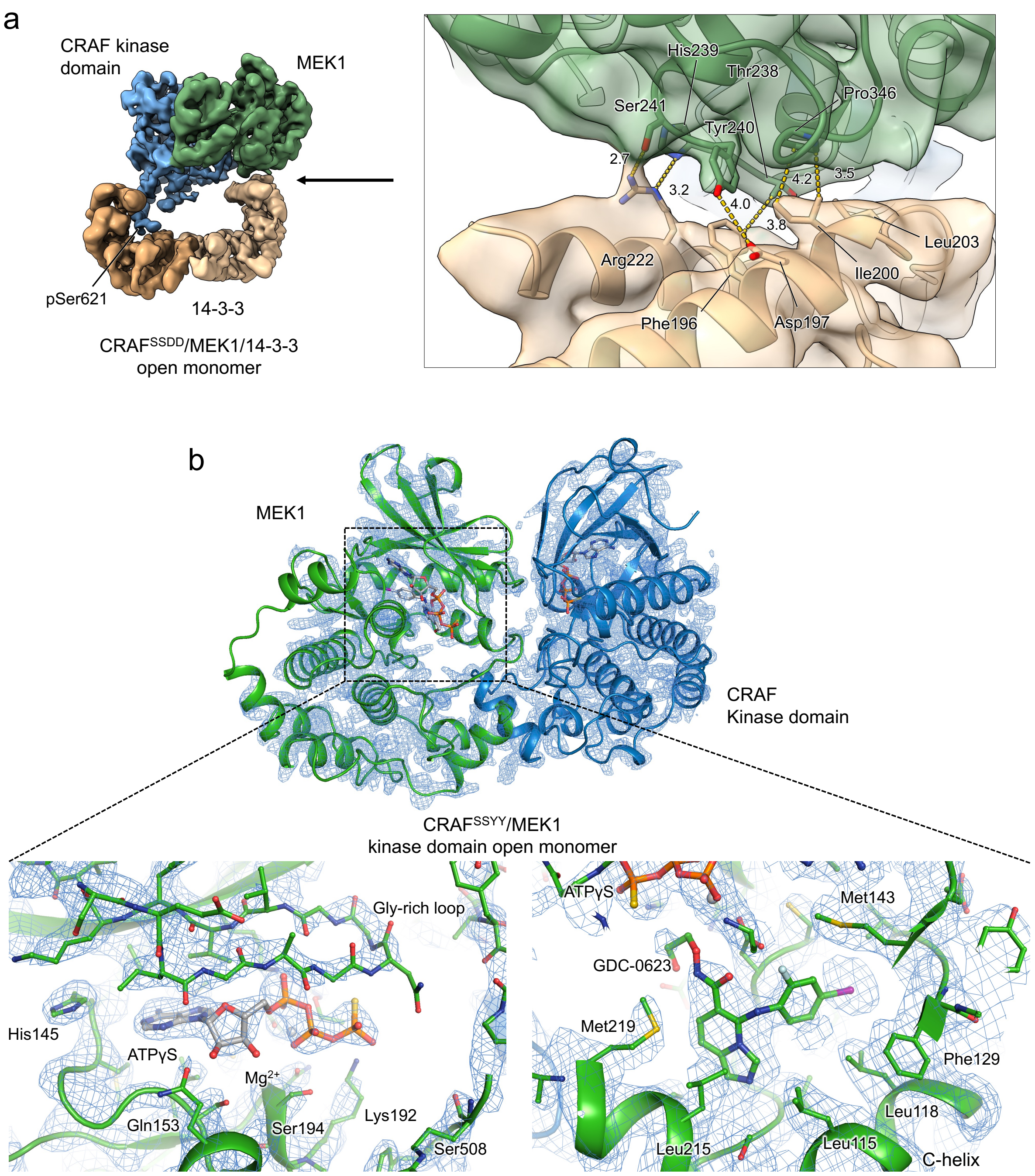

Supplementary Figure 10. Cryo-EM maps for the CRAF open monomers.

a, Overall cryo-EM map of the CRAF^{SSDD}/MEK1/14-3-3 open monomer. A close-up view of the MEK1–14-3-3 interface is shown in the direction indicated by the arrow. MEK1 is shown in green and 14-3-3 in tan. Residues at the interface are displayed as sticks, with contact distances labeled.

b, Overall view of the CRAF^{SSYY}/MEK1 kinase domain open monomer shown in ribbon representation with MEK1 colored green and CRAF colored blue. Close-up view of the MEK1 ATP-site showing density for the bound analog ATPγS. **c**, Close-up view of the MEK1 allosteric site showing density for the bound inhibitor GDC-0623.

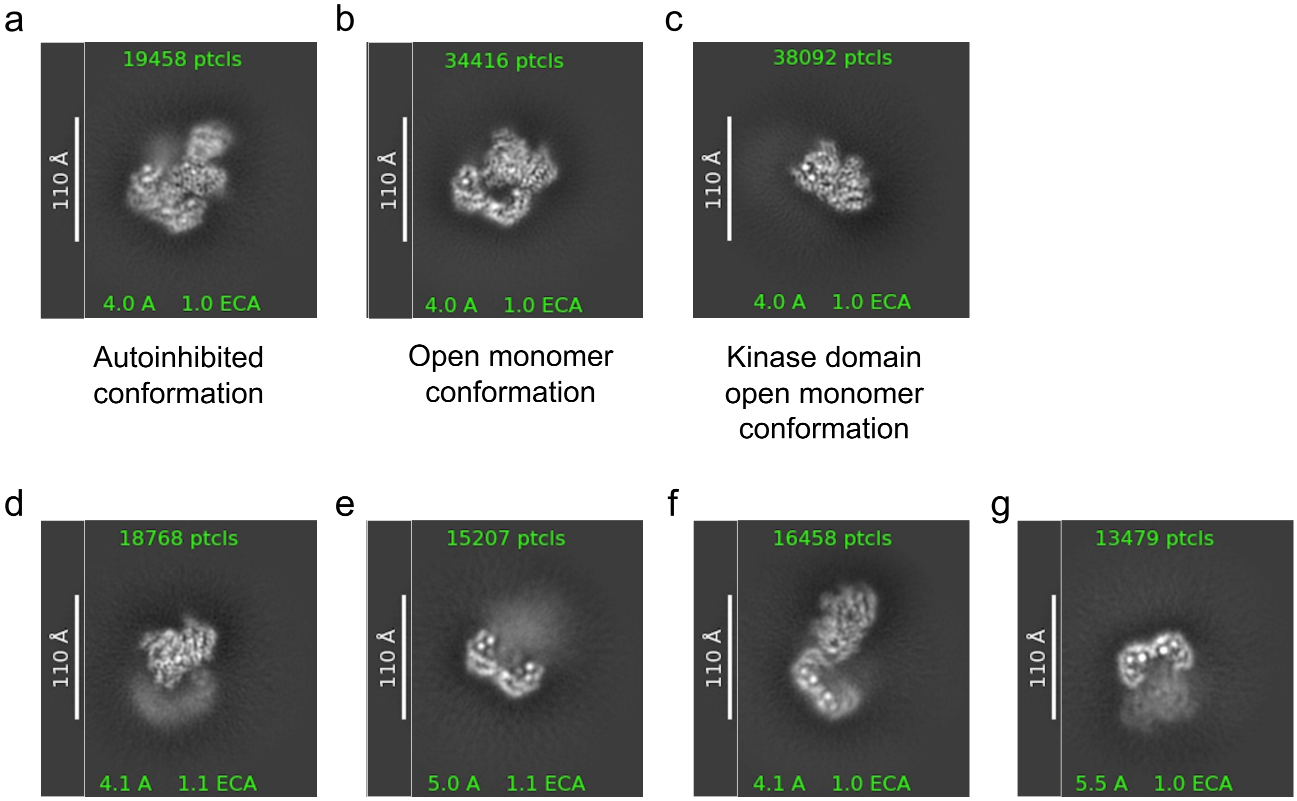

Supplementary Figure 11. Representative 2D class averages from the CRAF^{SSYY}/MEK/14-3-3 cryo-EM dataset.

a–c, Representative 2D class averages corresponding to the autoinhibited conformation (**a**), the open monomer conformation (**b**), and the kinase domain open monomer conformation (**c**). In **a**, note the blurred density in the upper left region of the particle which corresponds to the poorly ordered RBD, which we were not able to model. **d–g**, 2D class averages that illustrate the variability in orientations between the 14-3-3 dimer and CRAF/MEK1 kinase domain module in the open monomer state. In **d,** particles aligned based on CRAF/MEK1 kinase domain, but the 14-3-3 dimer appears smeared, indicating a degree of flexibility between the kinase module and the 14-3-3. In **e**, particles aligned based on the 14-3-3 dimer, but the CRAF/MEK1 kinase domains appear smeared. **f**, The 14-3-3 and CRAF/MEK1 kinase domains adopt a defined relative orientation, but one that is different from that in panel b. We were not able to obtain a 3D reconstruction for this configuration of the open monomer. **g**, Particles aligned based on the 14-3-3 dimer, but only a subset of the particles have a defined relative orientation of the CRAF/MEK1 kinase region, resulting in weak and blurred secondary structure features in this region. Each panel includes a scale bar (110 Å) and values for the number of particles (ptcls) contributing to each class average, resolution (Å), and the estimated CTF amplitude (ECA).

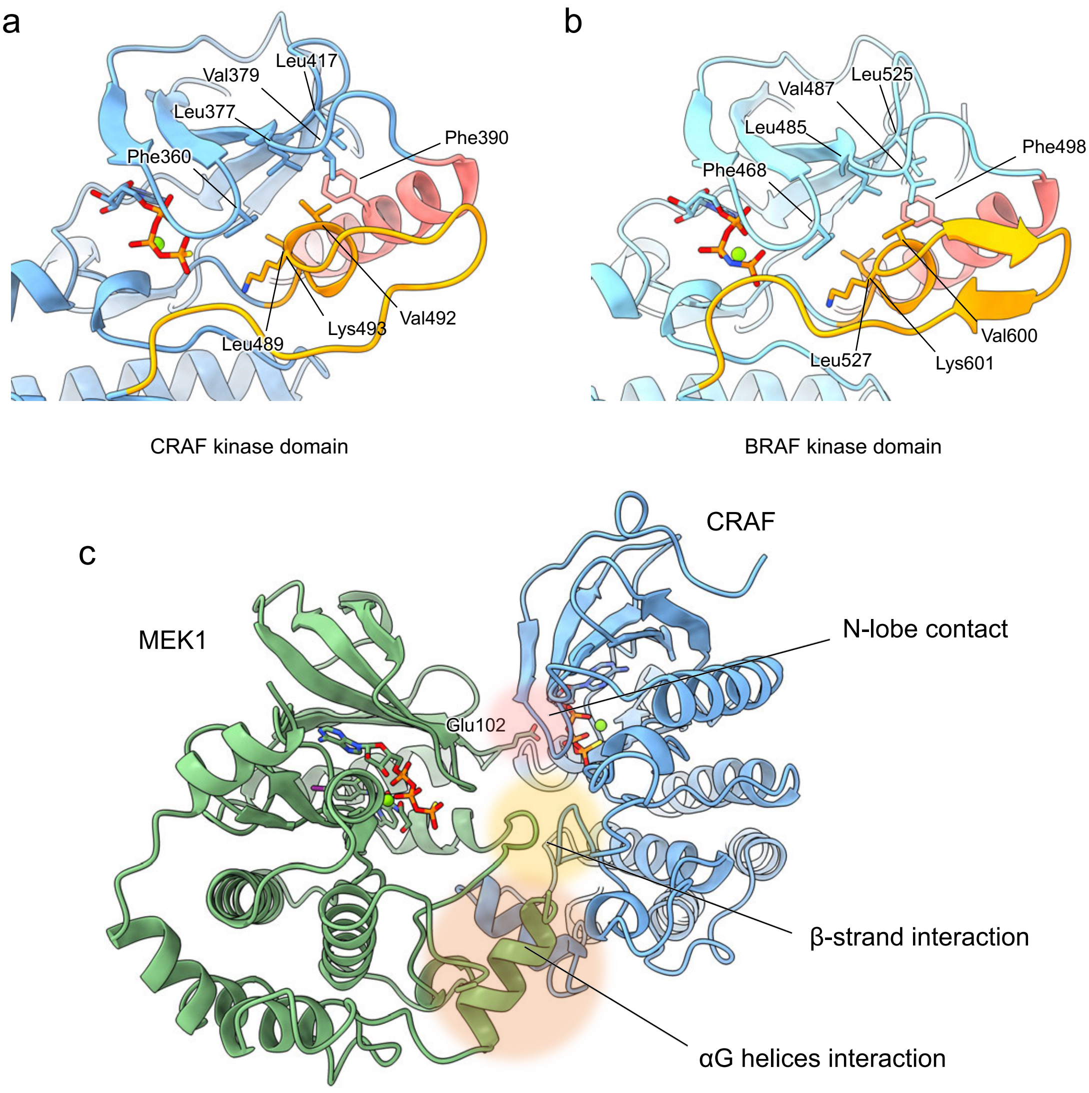

Supplementary Figure 12. The conserved autoinhibited conformation of the RAF kinase domain.

a and **b**, The inactive conformation of the CRAF kinase domain (panel **a**, drawn from the kinase domain open monomer structure) is closely similar to that of BRAF (panel **b**, drawn from PDB entry 6PP9). In both structures, the C-helix (pink) is positioned in an outward, inactive conformation, stabilized by the inhibitory turn within the activation segment (orange). Sidechains of selected residues are shown in stick form. Valine 492 in the inhibitory turn in CRAF corresponds to V600 in BRAF, the site of the oncogenic V600E mutation, and is similarly situated in a highly hydrophobic environment. ATPγS is bound in the CRAF active site cleft, AMPPNP in BRAF active site cleft. **c**, Overall structure of CRAF/MEK1 kinase domain (drawn from the kinase domain open monomer structure). The CRAF and MEK1 kinase domains interact in three regions: an N-lobe contact in which Glu102 of MEK1 is positioned to hydrogen bond with the ribose of the bound nucleotide in CRAF, an antiparallel β-strand interaction between portions of the activation loops of each kinase, and a mostly hydrophobic packing between the αG helices of each kinase.

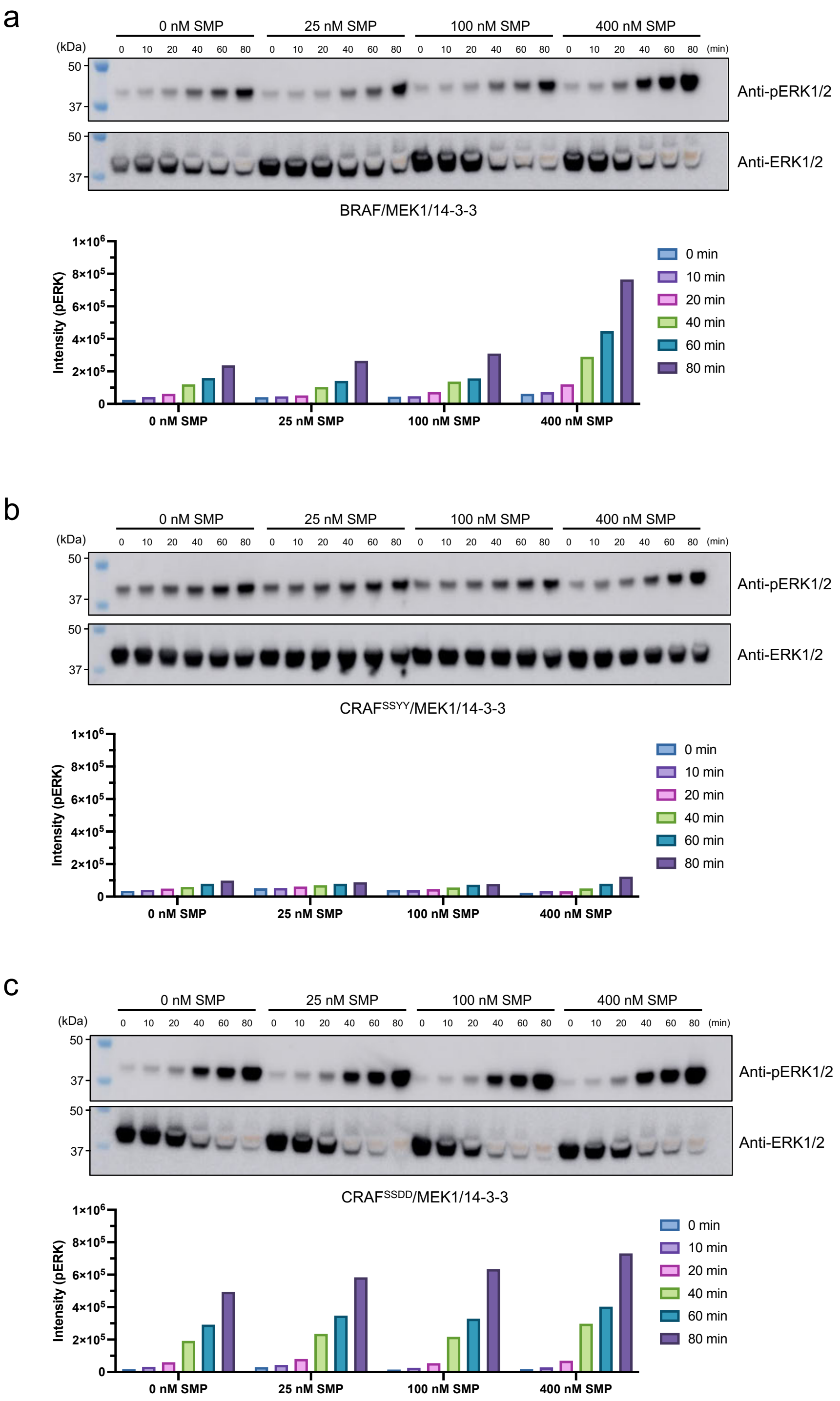

Supplementary Figure 13. Activation studies of RAF complexes upon dephosphorylation by the SHOC2 holophosphatase complex.

a-**c**, The activity of the indicated RAF complexes was measured in a cascade assay for ERK1/2 phosphorylation. BRAF/MEK1/14-3-3 (**a**), CRAF^{SSYY}/MEK1/14-3-3 (**b**), and CRAF^{SSDD}/MEK1/14-3-3 (**c**) complexes were incubated with increasing concentrations of the SHOC2 holophosphatase complex. Reactions were quenched by addition of a phosphatase inhibitor, then added to an assay mix containing 10 nM MEK1, 4 mM ERK2, and 1 mM ATP. The time course of phosphorylation of ERK2 was measured by western blotting with an anti-pERK1/2 antibody (upper panels). Total ERK2 levels were measured as a loading control using an anti-ERK1/2 antibody (lower panels). Note that although the anti-pERK1/2 blots were stripped before reprobing for total ERK, the very strong anti-pERK1/2 reactivity interfered with blotting for total ERK in some lanes. The intensity of phosphorylated ERK2 was quantified and plotted in the bottom panels.
