# Supplementary material for: Cryo-EM structures of CRAF/MEK1/14-3-3 complexes in autoinhibited and open-monomer states reveal features of RAF regulation
Source: Nat Commun. 2025 Sep 1;16:8150. doi: 10.1038/s41467-025-63227-2 (PMC12402067; doi:10.1038/s41467-025-63227-2)
Supplement: Supplementary file 2 — Reporting Summary [file 41467_2025_63227_MOESM2_ESM.pdf]

nature portfolio

Corresponding author(s): Michael J. Eck

Last updated by author(s): Jul 18, 2025

Reporting Summary

Nature Portfolio wishes to improve the reproducibility of the work that we publish. This form provides structure for consistency and transparency in reporting. For further information on Nature Portfolio policies, see our Editorial Policies and the Editorial Policy Checklist.

Statistics

For all statistical analyses, confirm that the following items are present in the figure legend, table legend, main text, or Methods section.

n/a	Confirmed	
☐	☒	The exact sample size (*n*) for each experimental group/condition, given as a discrete number and unit of measurement
☐	☒	A statement on whether measurements were taken from distinct samples or whether the same sample was measured repeatedly
☒	☐	The statistical test(s) used AND whether they are one- or two-sided<br>*Only common tests should be described solely by name; describe more complex techniques in the Methods section.*
☒	☐	A description of all covariates tested
☒	☐	A description of any assumptions or corrections, such as tests of normality and adjustment for multiple comparisons
☐	☒	A full description of the statistical parameters including central tendency (e.g. means) or other basic estimates (e.g. regression coefficient) AND variation (e.g. standard deviation) or associated estimates of uncertainty (e.g. confidence intervals)
☒	☐	For null hypothesis testing, the test statistic (e.g. *F*, *t*, *r*) with confidence intervals, effect sizes, degrees of freedom and *P* value noted<br>*Give P values as exact values whenever suitable.*
☒	☐	For Bayesian analysis, information on the choice of priors and Markov chain Monte Carlo settings
☒	☐	For hierarchical and complex designs, identification of the appropriate level for tests and full reporting of outcomes
☒	☐	Estimates of effect sizes (e.g. Cohen's *d*, Pearson's *r*), indicating how they were calculated

Our web collection on statistics for biologists contains articles on many of the points above.

Software and code

Policy information about availability of computer code

Data collection	Standard widely available software was used for, including EPU for Krios microscope and Falcon 4i detector.
Data analysis	Standard widely available software was used for structure determination, including CryoSPARC v4.5.3, crYOLO v1.9.9, Coot v0.9.8.92, and PHENIX v1.21.1-5286. References are provided in the Reference section.

For manuscripts utilizing custom algorithms or software that are central to the research but not yet described in published literature, software must be made available to editors and reviewers. We strongly encourage code deposition in a community repository (e.g. GitHub). See the Nature Portfolio guidelines for submitting code & software for further information.

Data

Policy information about availability of data

All manuscripts must include a data availability statement. This statement should provide the following information, where applicable:

- Accession codes, unique identifiers, or web links for publicly available datasets
- A description of any restrictions on data availability
- For clinical datasets or third party data, please ensure that the statement adheres to our policy

The cryo-EM maps of the CRAFSSYY/MEK1SASA/14-3-3 complex in the autoinhibited conformation and kinase domain open monomer conformation were deposited to the EM Data Bank (https://www.ebi.ac.uk/emdb/) under accession codes EMD-48397 and EMD-48399, respectively. Cryo-EM maps for the CRAFSSDD/MEK1SASA/14-3-3 complex in the open monomer conformation and kinase domain open monomer conformation were deposited to the EM Data Bank under

accession codes EMD-48401 and EMD-48402, respectively.
Atomic models for the CRAFSSYY/MEK1SASA/14-3-3 complex in the autoinhibited conformation and kinase domain open monomer conformation were deposited to the Protein Data Bank (PDB) and are available at www.rcsb.org under accession codes 9MMP and 9MMQ. Atomic models for the CRAFSSDD/MEK1SASA/14-3-3 complex in the open monomer conformation and kinase domain open monomer conformations were deposited to the PDB under accession codes 9MMR and 9MMS, respectively. Detailed information for all maps and models generated in this work is provided in Supplementary Tables 1 and 2.

Research involving human participants, their data, or biological material

Policy information about studies with human participants or human data. See also policy information about sex, gender (identity/presentation), and sexual orientation and race, ethnicity and racism.

Reporting on sex and gender	N/A
Reporting on race, ethnicity, or other socially relevant groupings	N/A
Population characteristics	N/A
Recruitment	N/A
Ethics oversight	N/A

Note that full information on the approval of the study protocol must also be provided in the manuscript.

Field-specific reporting

Please select the one below that is the best fit for your research. If you are not sure, read the appropriate sections before making your selection.

☒ Life sciences ☐ Behavioural & social sciences ☐ Ecological, evolutionary & environmental sciences

For a reference copy of the document with all sections, see nature.com/documents/nr-reporting-summary-flat.pdf

Life sciences study design

All studies must disclose on these points even when the disclosure is negative.

Sample size	Single particle reconstruction for cryo-EM was performed from four data sets.
Data exclusions	No excluded data.
Replication	The experiments regarding the Figure 1d, Figure 4a-c upper panels, Figure 5b, Supplementary Figure 2e were perfored three times. The experiments regarding the Figure 4 a-c lower panels, Figure 5a, and Supplementary Figure 13 were performed two times. The experiments regarding the Supplementary Figure 2 a-d were performed once.
Randomization	Randomization is not neccessary or appropriate in these biochemical and biophysical experiments.
Blinding	Blinding is not neccessary or appropriate in these biochemical and biophysical experiments.

Reporting for specific materials, systems and methods

We require information from authors about some types of materials, experimental systems and methods used in many studies. Here, indicate whether each material, system or method listed is relevant to your study. If you are not sure if a list item applies to your research, read the appropriate section before selecting a response.

Materials & experimental systems

n/a	Involved in the study	
☐	☒	Antibodies
☐	☒	Eukaryotic cell lines
☒	☐	Palaeontology and archaeology
☒	☐	Animals and other organisms
☒	☐	Clinical data
☒	☐	Dual use research of concern
☒	☐	Plants

Methods

n/a	Involved in the study	
☒	☐	ChIP-seq
☒	☐	Flow cytometry
☒	☐	MRI-based neuroimaging

Antibodies

Antibodies used	Anti-ERK(Cell Signaling Technology,#9102), RRID:AB_330744,1:1000dilution<br>Anti-pERK (Cell Signaling Technology, #45899), RRID:AB_2315112, 1:1000 dilution<br>Anti-CRAF (Cell Signaling Technology, #53745), RRID:AB_2799444, 1:1000 dilution<br>Anti-CRAF pS259 (Cell Signaling Technology, #9421), RRID:AB_10976055, 1:1000 dilution<br>Anti-CRAF pS338 (Cell Signaling Technology, #9427), RRID:AB_2067317, 1:1000 dilution<br>Anti-CRAF pY341 (abcam, #ab59223), RRID:AB_946320, 1:1000 dilution<br>Anti-BRAF (Cell Signaling Technology, #14814), RRID:AB_2750887, 1:1000 dilution
Validation	Listed RRID for each antibody above.

Eukaryotic cell lines

Policy information about cell lines and Sex and Gender in Research

Cell line source(s)	GibcoTM Sf9 cells in Sf-900TM ll SFM (ThermoFisher Scientific, Cat.no.11496016)
Authentication	Cells were purchased from ThermoFisher and not independently authenticated.
Mycoplasma contamination	Vendor (ThermoFisher)'s producct quality confirms negative.
Commonly misidentified lines<br>(See ICLAC register)	N/A

Plants

Seed stocks	N/A
Novel plant genotypes	N/A
Authentication	N/A
