# Supplementary material for: Cryo-EM structures of CRAF/MEK1/14-3-3 complexes in autoinhibited and open-monomer states reveal features of RAF regulation
Source: Nat Commun. 2025 Sep 1;16:8150. doi: 10.1038/s41467-025-63227-2 (PMC12402067; doi:10.1038/s41467-025-63227-2)

Fig. 4

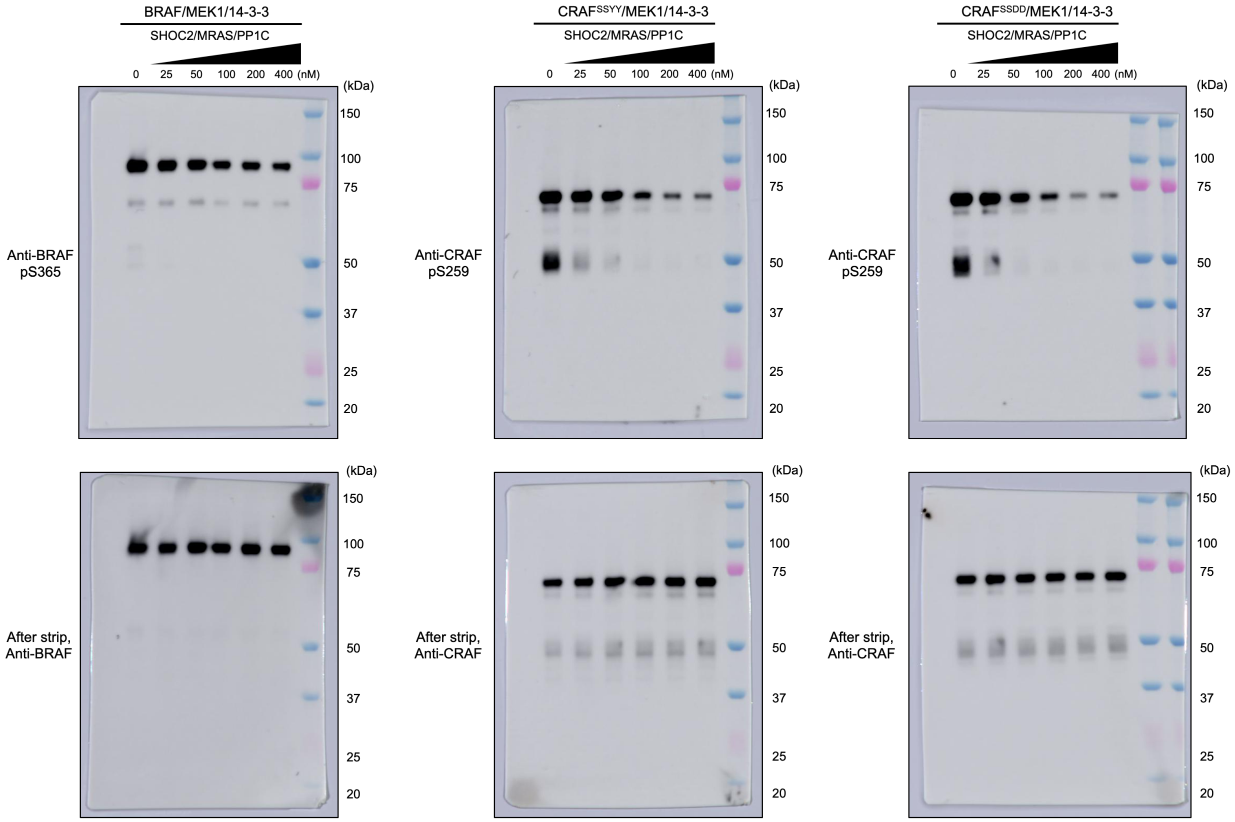

BRAF/MEK1/14-3-3
SHOC2/MRAS/PP1C
0 25 50 100 200 400 (nM)
CRAF^{SSYY}/MEK1/14-3-3
SHOC2/MRAS/PP1C
0 25 50 100 200 400 (nM)
CRAF^{SSDD}/MEK1/14-3-3
SHOC2/MRAS/PP1C
0 25 50 100 200 400 (nM)
(kDa)
150
100
75
50
37
25
20
Anti-BRAF pS365
Anti-CRAF pS259
Anti-CRAF pS259
After strip, Anti-BRAF
After strip, Anti-CRAF
After strip, Anti-CRAF

Fig 5.

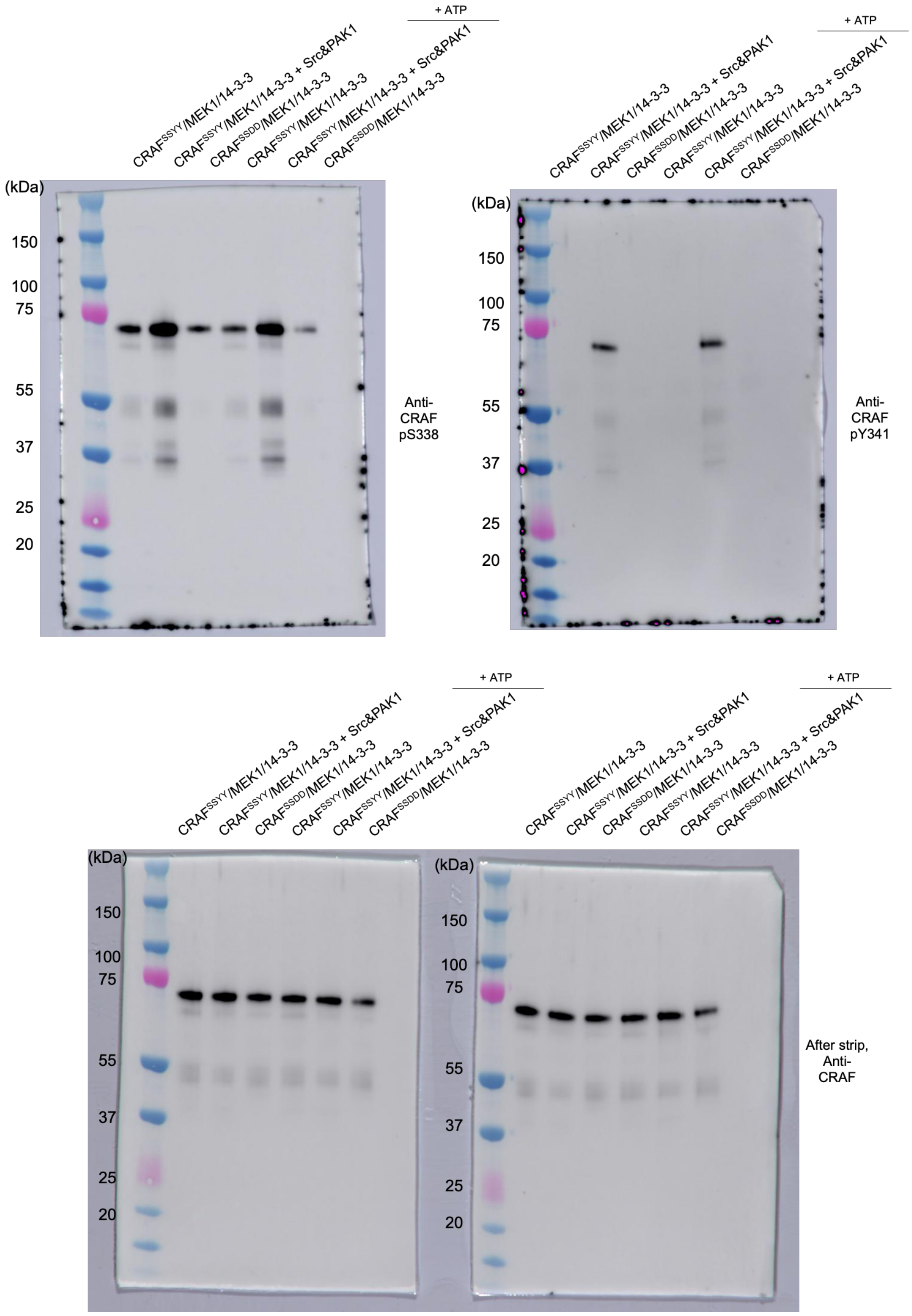
+ ATP
CRAF^{SSYY}/MEK1/14-3-3
CRAF^{SSYY}/MEK1/14-3-3 + Src&PAK1
CRAF^{SSDD}/MEK1/14-3-3
CRAF^{SSYY}/MEK1/14-3-3
CRAF^{SSYY}/MEK1/14-3-3 + Src&PAK1
CRAF^{SSDD}/MEK1/14-3-3
(kDa)
150
100
75
55
37
25
20
Anti-CRAF pS338
+ ATP
CRAF^{SSYY}/MEK1/14-3-3
CRAF^{SSYY}/MEK1/14-3-3 + Src&PAK1
CRAF^{SSDD}/MEK1/14-3-3
CRAF^{SSYY}/MEK1/14-3-3
CRAF^{SSYY}/MEK1/14-3-3 + Src&PAK1
CRAF^{SSDD}/MEK1/14-3-3
(kDa)
150
100
75
55
37
25
20
Anti-CRAF pY341
+ ATP
CRAF^{SSYY}/MEK1/14-3-3
CRAF^{SSYY}/MEK1/14-3-3 + Src&PAK1
CRAF^{SSDD}/MEK1/14-3-3
CRAF^{SSYY}/MEK1/14-3-3
CRAF^{SSYY}/MEK1/14-3-3 + Src&PAK1
CRAF^{SSDD}/MEK1/14-3-3
(kDa)
150
100
75
55
37
25
20
+ ATP
CRAF^{SSYY}/MEK1/14-3-3
CRAF^{SSYY}/MEK1/14-3-3 + Src&PAK1
CRAF^{SSDD}/MEK1/14-3-3
CRAF^{SSYY}/MEK1/14-3-3
CRAF^{SSYY}/MEK1/14-3-3 + Src&PAK1
CRAF^{SSDD}/MEK1/14-3-3
(kDa)
150
100
75
55
37
25
20
After strip, Anti-CRAF

Supplementary Fig. 2a

CRAF^{SSYY}/MEK1/14-3-3

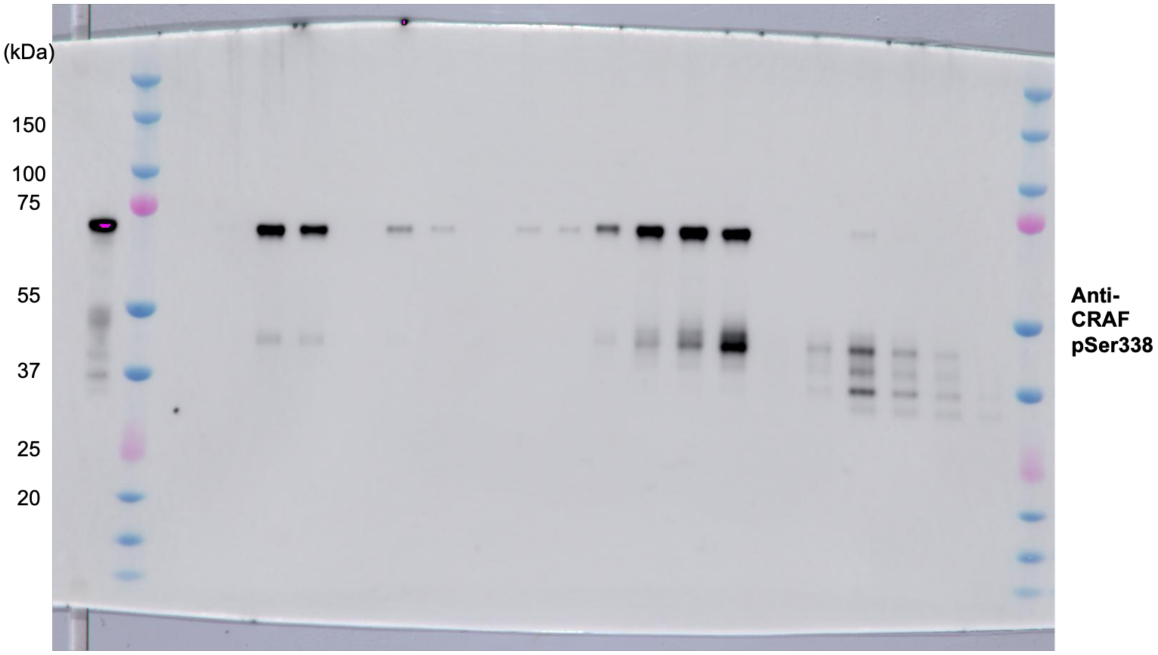

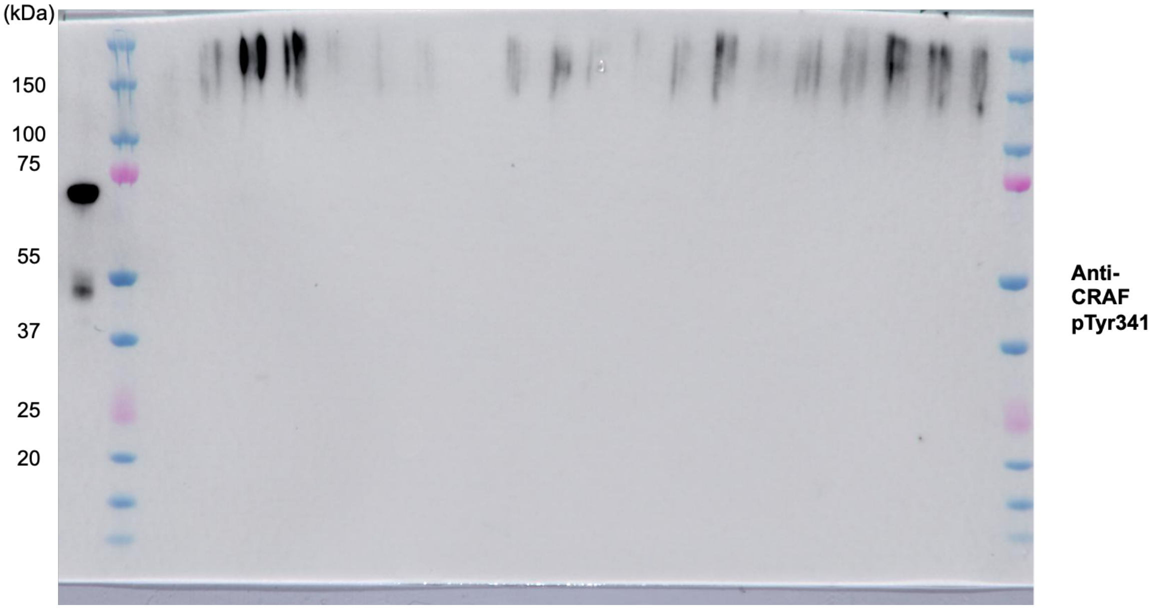

Supplementary Fig. 2b

CRAF^{SSDD}/MEK1/14-3-3

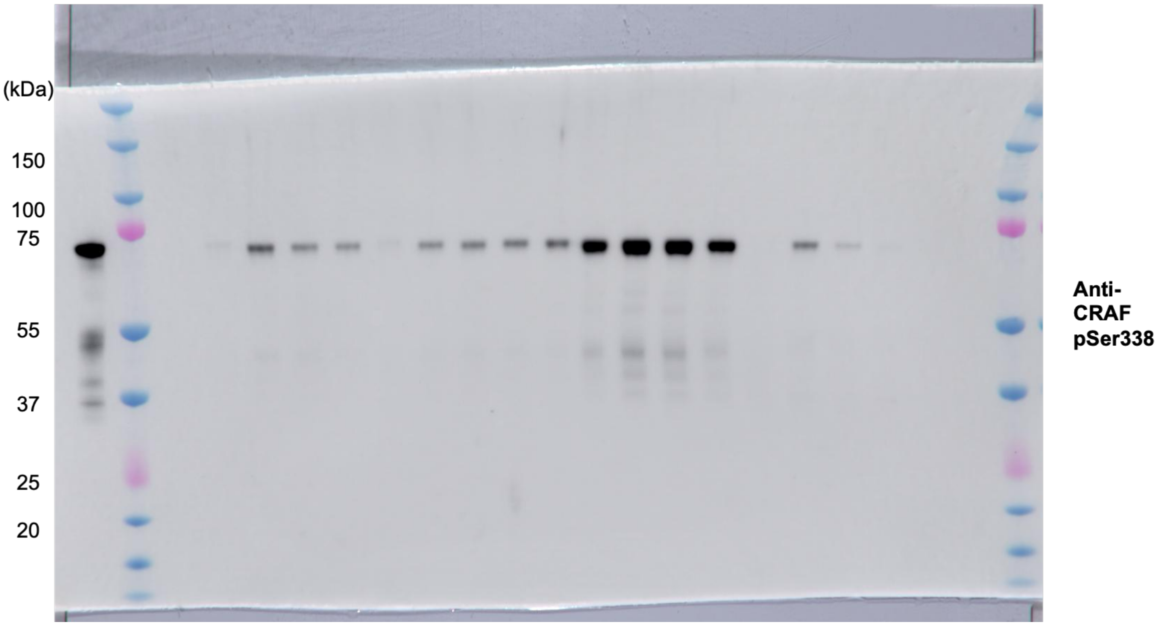

Supplementary Fig. 2c

CRAF^{SSYY}/MEK1/14-3-3 + Src&PAK1

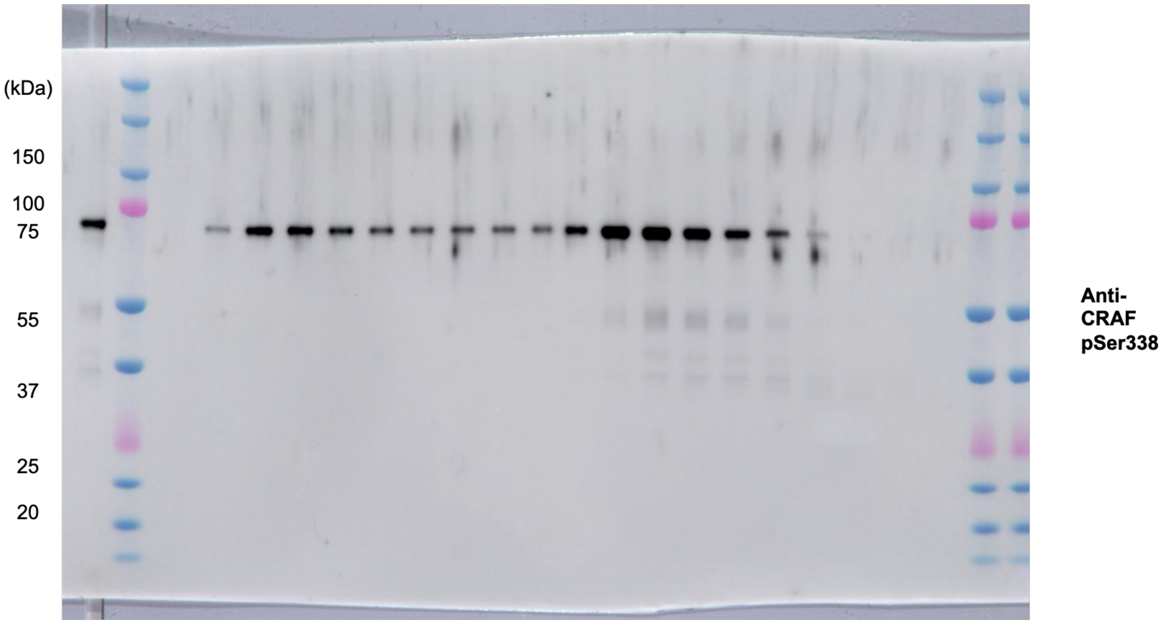

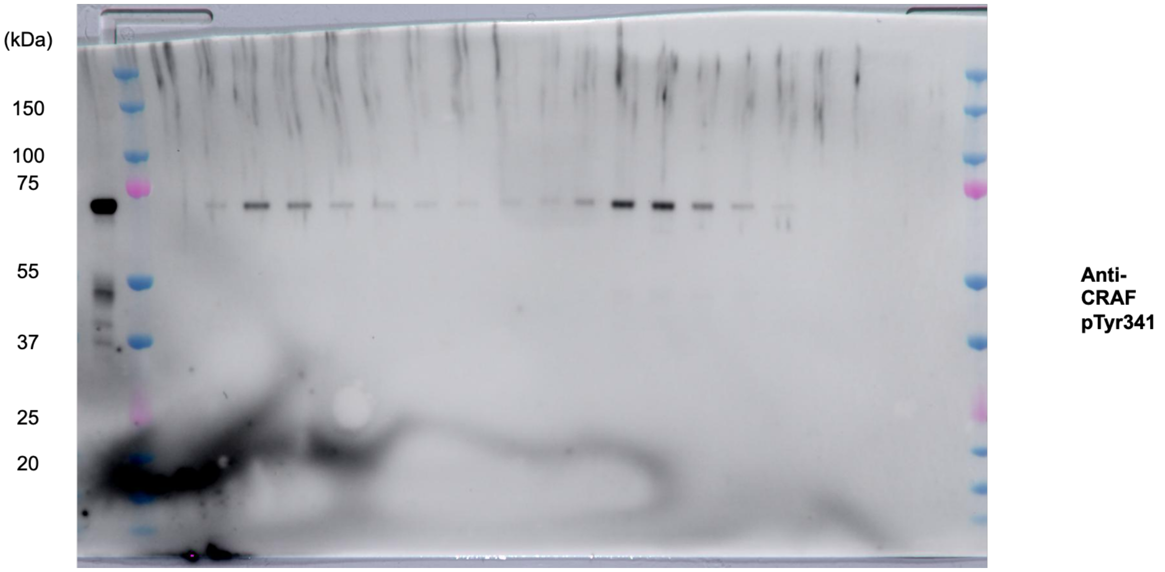

Supplementary Fig. 10a

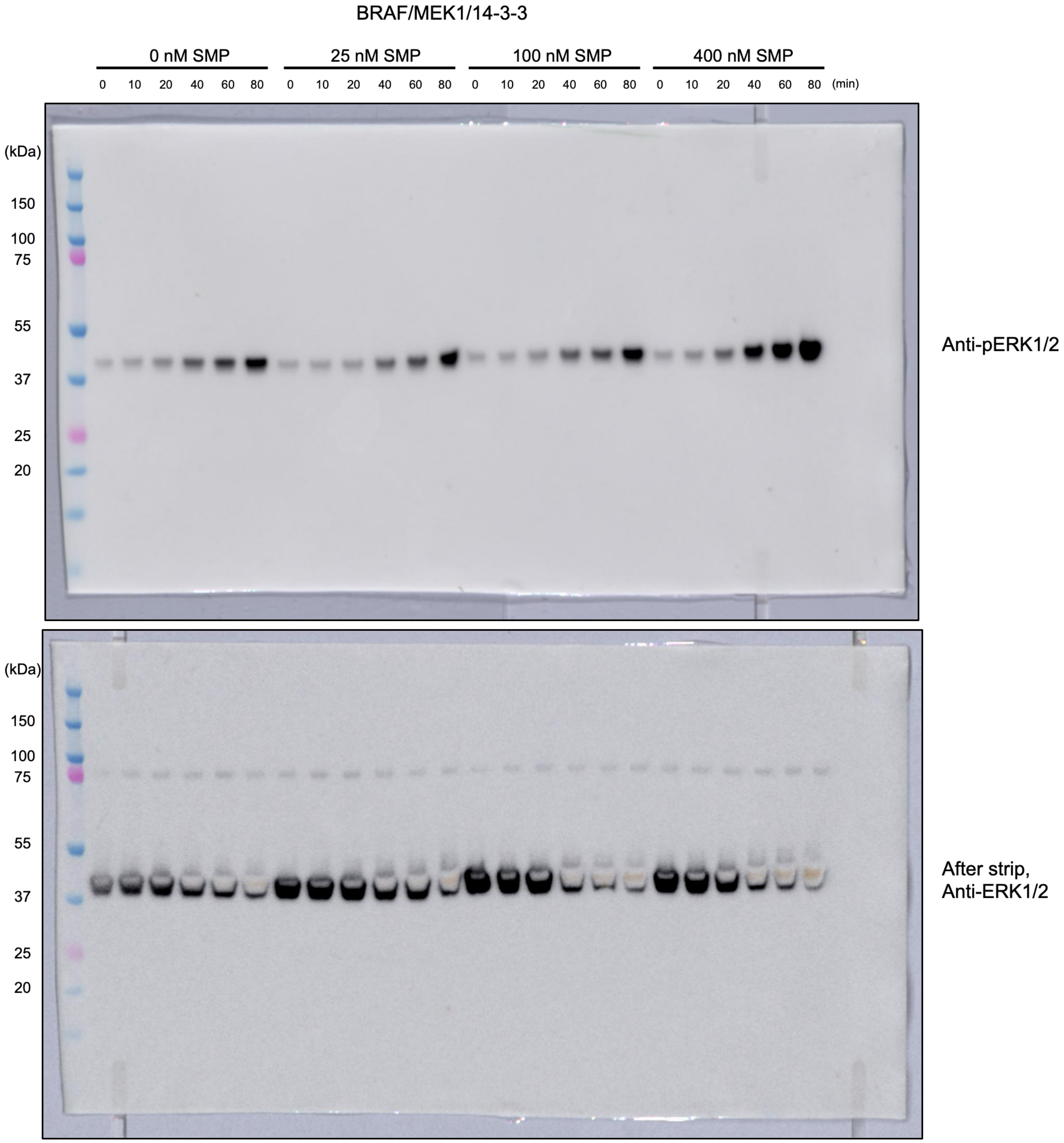

Supplementary Fig. 10b

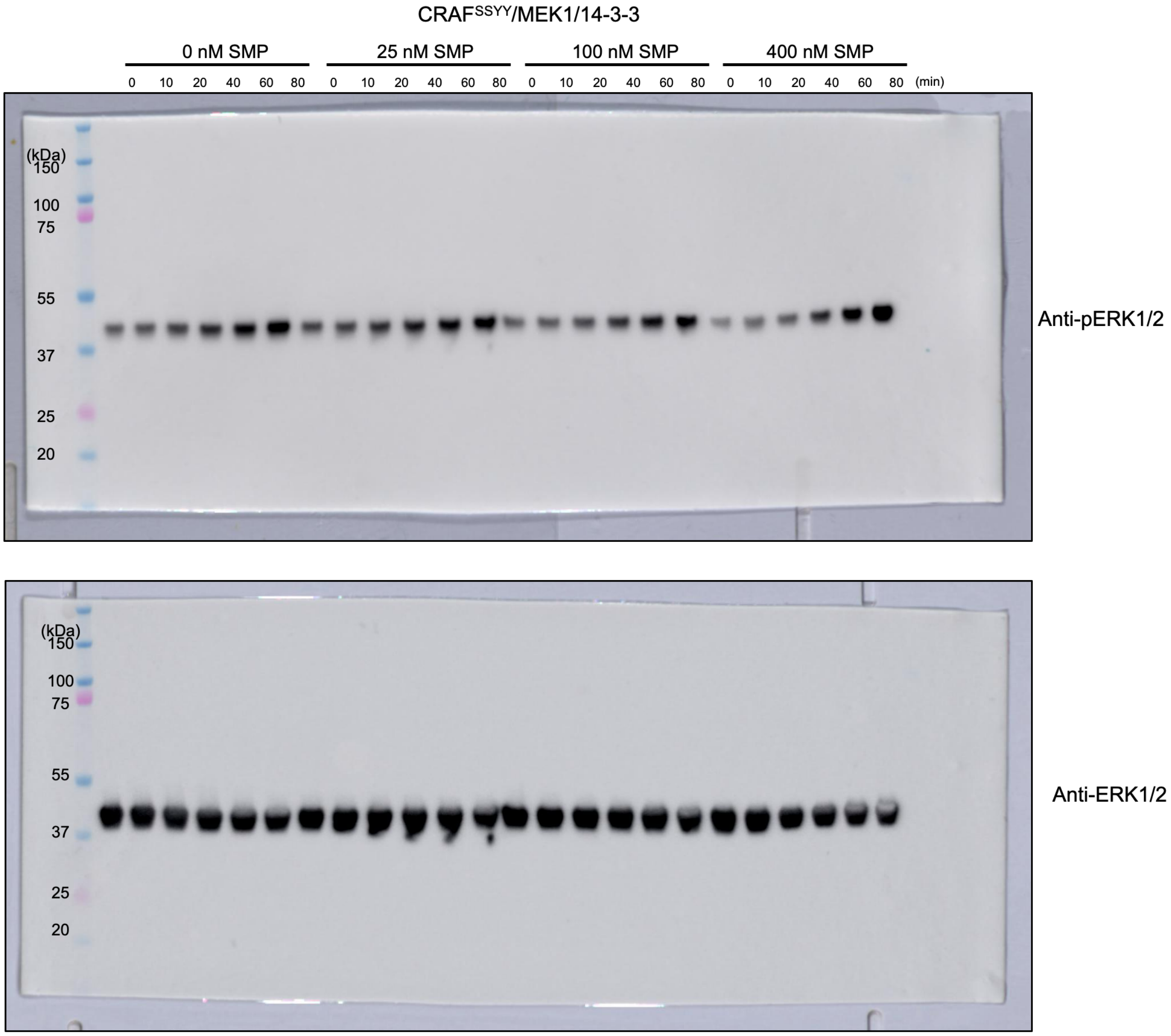

Supplementary Fig. 10c

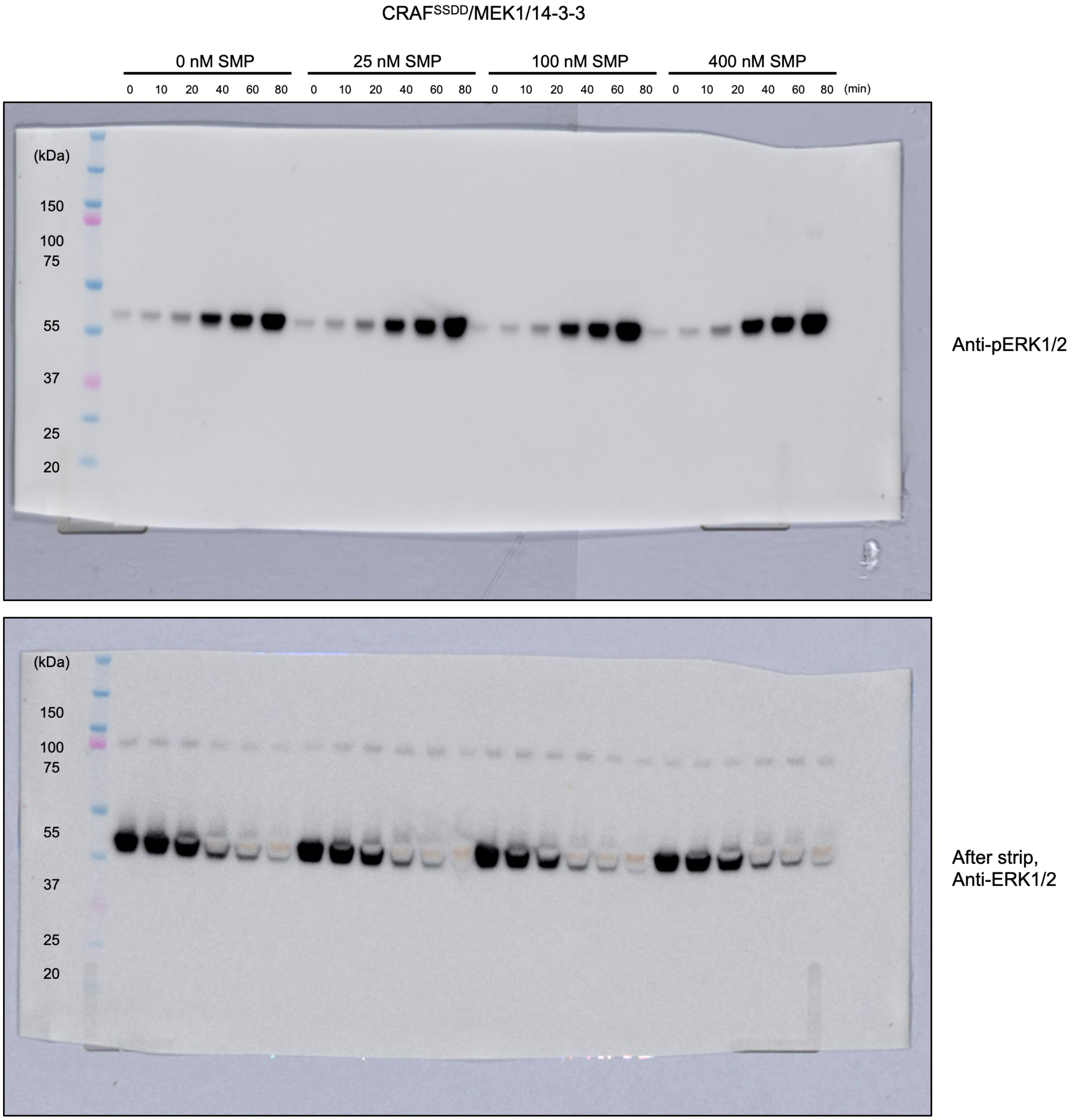

Supplement: Supplementary file 4 — Source Data [file 41467_2025_63227_MOESM4_ESM.zip › Source-data-files/Western_blot_Raw_images.pdf]
