# Supplementary material for: Cryo-EM structures of CRAF/MEK1/14-3-3 complexes in autoinhibited and open-monomer states reveal features of RAF regulation
Source: Nat Commun. 2025 Sep 1;16:8150. doi: 10.1038/s41467-025-63227-2 (PMC12402067; doi:10.1038/s41467-025-63227-2)
Supplement: Supplementary file 3 — Transparent Peer Review file [file 41467_2025_63227_MOESM3_ESM.pdf]

nature portfolio

Peer Review File

Cryo-EM structures of CRAF/MEK1/14-3-3 complexes in autoinhibited and open-monomer states reveal features of RAF regulation.

Corresponding Author: Dr Michael Eck

This manuscript has been previously reviewed at another journal. This document only contains information relating to versions considered at Nature Communications.

Version 0:

Reviewer comments:

Reviewer #1

(Remarks to the Author)
The authors addressed all my concerns and I think the work should be published.

Reviewer #2

(Remarks to the Author)
The authors have satisfactorily addressed all my previous comments.

Reviewer #3

(Remarks to the Author)
The authors have done well to address comments from the earlier review. I have no further questions. Congrats to the authors on an excellent manuscript.

Open Access This Peer Review File is licensed under a Creative Commons Attribution 4.0 International License, which permits use, sharing, adaptation, distribution and reproduction in any medium or format, as long as you give appropriate credit to the original author(s) and the source, provide a link to the Creative Commons license, and indicate if changes were made.
In cases where reviewers are anonymous, credit should be given to 'Anonymous Referee' and the source.
The images or other third party material in this Peer Review File are included in the article's Creative Commons license, unless indicated otherwise in a credit line to the material. If material is not included in the article's Creative Commons license and your intended use is not permitted by statutory regulation or exceeds the permitted use, you will need to obtain permission directly from the copyright holder.
To view a copy of this license, visit https://creativecommons.org/licenses/by/4.0/
